# Effect of Pulsed Low-Intensity Ultrasonography on Symptom Relief and Tibiofemoral Articular Cartilage Thickness Among Veterans Affairs Enrollees With Knee Osteoarthritis: A Randomized Clinical Trial

**DOI:** 10.1001/jamanetworkopen.2022.0632

**Published:** 2022-03-08

**Authors:** Allen D. Sawitzke, Christopher G. Jackson, Kimberly Carlson, Marcel D. Bizien, Mathew Leiner, Domenic J. Reda, Tom Sindowski, Christopher Hanrahan, Richard G. Spencer, C. Kent Kwoh, Susan J. Lee, Kalli Hose, Lisa Robin, Donna W. Cain, Meredith D. Taylor, Neal Bangerter, Martha Finco, Daniel O. Clegg

**Affiliations:** 1 Department of Medicine, University of Utah, Salt Lake City; 2 George E. Wahlen Department of Veterans Affairs Medical Center, Salt Lake City, Utah; 3 Edward Hines Junior VA Hospital Cooperative Studies Program Coordinating Center, Hines, Illinois; 4 VA Cooperative Studies Program, Clinical Research Pharmacy Coordinating Center, Albuquerque, New Mexico; 5 School of Pharmacy, University of New Mexico, Albuquerque, New Mexico; 6 National Institutes of Health/National Institute on Aging, Laboratory of Clinical Investigation, Baltimore, Maryland; 7 University of Arizona Arthritis Center, University of Arizona, Tucson; 8 VA San Diego Healthcare System, San Diego, California; 9 Department of Medicine, San Diego VA Medical Center, San Diego, California; 10 Department of Electrical & Computer Engineering, Brigham Young University, Provo, Utah; 11 Department of Radiology, University of Utah, Salt Lake City; 12 Department of Orthopedics, University of Utah, Salt Lake City; 13 Department of Bioengineering, Imperial College London, London, United Kingdom

## Abstract

**Importance:**

Osteoarthritis (OA) is a major cause of disability in the US, with no approved treatments to slow progression, but animal models suggest that pulsed low-intensity ultrasonography (PLIUS) may promote cartilage growth.

**Objective:**

To evaluate the efficacy of PLIUS in providing symptom reduction and decreased loss of tibiofemoral cartilage thickness in patients with knee OA.

**Design, Setting, and Participants:**

A phase 2A, sham-controlled, parallel, double-blind randomized clinical trial was conducted at 2 Veterans Affairs hospitals in Salt Lake City, Utah, and San Diego, California, from May 22, 2015, to January 31, 2019. Data were analyzed from June 27, 2020, to October 20, 2020. Participants recruited through the US Department of Veterans Affairs (N = 132) with clinical and radiographic evidence of early knee OA were randomly assigned to receive PLIUS or a sham device, self-administered for 20 minutes daily over the medial compartment of the knee. All enrollees participated in a 4-week prerandomization sham run-in period, followed by a 48-week treatment period. Randomization was stratified by study site and Kellgren-Lawrence grades 1 (n = 15), 2 (n = 51), and 3 (n = 66).

**Intervention:**

Participants either received 48 weeks of PLIUS or sham ultrasonography.

**Main Outcomes and Measures:**

The trial incorporated 2 coprimary outcomes: symptomatic improvement assessed by Outcome Measures in Rheumatology Clinical Trials–Osteoarthritis Research Society International Responder Criteria (ie, met if either >50% improvement in pain and function with at least a 20% absolute improvement of at least 2 of the following 3 factors: improvement by at least 20% [pain, function, and patient global assessment] with at least a 10-mm absolute improvement), and cartilage preservation assessed as change in central medial femoral condyle cartilage thickness by magnetic resonance imaging. Intention-to-treat analysis was used.

**Results:**

The mean (SD) participant age was 63.6 (10.7) years and 119 were men (90.2%). The mean (SD) duration of OA symptoms was 13.4 (12.3) years. In the PLIUS group, 70.4% (95% CI, 58.2%-82.6%) of the participants experienced symptomatic improvement, compared with 67.3% (95% CI, 54.9%-79.7%) of participants in the sham group (*P* = .84); there was no statistically significant difference in response rates between the treatment groups, and the between-group rate difference of 3.1% (95% CI, –14.3% to 20.5%) did not meet the predefined 10% threshold for clinically significant symptomatic improvement from application of PLIUS. At 48 weeks of treatment, central medial femoral condyle cartilage thickness decreased by a mean (SD) of 73.8 (168.1) μm in the PLIUS group and by 42.2 (297.0) μm in the sham group. This 48-week mean change between the 2 groups did not reach statistical significance (*P* = .44), and the between-group 48-week difference of –31.7 μm (95% CI, –129.0 μm to 65.7 μm) did not meet the predefined threshold. There were 99 nonserious adverse events in the PLIUS group and 89 in the sham group during the trial. No serious adverse events were deemed related to the study device.

**Conclusions and Relevance:**

PLIUS, as implemented in this study, demonstrated neither symptomatic benefit nor a decrease in loss of tibiofemoral cartilage thickness in knee OA.

**Trial Registration:**

ClinicalTrials.gov Identifier: NCT02034409

## Key Points

**Question:**

Does 48 weeks of pulsed low-intensity ultrasonography (PLIUS) provide therapeutic benefit for patients with idiopathic knee osteoarthritis?

**Findings:**

In this randomized clinical trial of 132 adults with symptomatic and radiographic idiopathic knee osteoarthritis, no statistically significant difference was found between PLIUS and sham therapies for clinical symptoms or tibiofemoral cartilage thickness, the coprimary outcomes.

**Meaning:**

PLIUS as applied in this trial was not effective in ameliorating symptoms or slowing cartilage loss associated with idiopathic knee osteoarthritis.

## Teaser

This randomized clinical trial evaluates the use of pulsed low-intensity ultrasonography for symptom relief and cartilage loss in individuals with osteoarthritis of the knee.

## Introduction

Osteoarthritis (OA) affects almost 27 million individuals in the US with an estimated net cost of more than $80 billion per year.^1,2^ Knee OA is particularly common with advancing age, with 30% of individuals older than 45 years having radiographic changes and approximately half of those being symptomatic.^3^ It is the leading cause of lower extremity disability in the US and is the most frequent indication for total knee replacement.^4^ By 2030, an estimated 60 million people in the US will be affected.^5^ Medical management at present is directed only at symptom relief^6^ and, at best, has marginal long-term efficacy. Osteoarthritis is known to occur disproportionately in members of the armed services,^7^ with the burden of disease evidenced by the fact that within the Veterans Affairs system, total knee replacement is a very common elective surgical procedure.

Despite substantial progress in understanding the pathogenesis of OA, no disease-modifying interventions to slow or stop its progression have been given regulatory approval.^3,8^ Several surgical techniques intended to repair, regenerate, or replace damaged cartilage have been attempted, including microfracture,^9^ autograft and allograft transplants,^10^ and autologous chondrocyte implantation^11^; however, long-term outcomes have been disappointing for all of these procedures.

Pulsed low-intensity ultrasonography (PLIUS) has long been used for fracture healing^12,13^ and is also known to increase cartilage matrix production.^14,15^ Both in vitro and animal studies suggest the potential for PLIUS in promoting cartilage growth^16,17,18,19,20^ through molecular-level signaling,^21^ increased aggrecan and collagen gene expression,^18^ related protein production in cell culture systems,^17^ and improved cartilage tissue integrity in animal OA models.^16,20,22^ PLIUS has also been found to attenuate cartilage degradation in the guinea pig model of idiopathic age-associated OA.^23^

In preliminary human studies, PLIUS has resulted in reduced OA symptoms,^24,25,26^ and a disease-modifying potential for PLIUS has been suggested by a post hoc subgroup analysis of an additional human study.^27^ There have been indicators of a positive effect on cartilage repair.^28,29^ The present sham-controlled, parallel, double-blind, phase 2A randomized clinical trial was undertaken as a pilot to extend these preliminary findings and determine whether a subsequent phase 2b or phase 3 trial of PLIUS for OA therapy would be warranted.

## Methods

### Participants

Study participants were beneficiaries of the Department of Veterans Affairs with a previous service history, recruited from Salt Lake City, Utah, or San Diego, California, VA research centers from May 22, 2015, until January 31, 2019. Data were analyzed from June 27, 2020, to October 20, 2020. Demographic data collected included age, sex, race, and ethnicity. Data on race were obtained as required by the funding agency but were not included in the analysis owing to the small numbers. Inclusion criteria comprised age of at least 40 years, clinical symptoms of OA defined by knee pain for at least 6 months and on most days during the month preceding study entry, and radiographic evidence of OA defined by Kellgren-Lawrence grade (KLG) (1: doubtful narrowing of joint space, possible osteophytic lipping; 2: definite osteophytes and possible narrowing of joint space; and 3: moderate multiple osteophytes, definite narrowing of joint space, some sclerosis, and possible deformity) evaluated from posterior-anterior weightbearing knee radiographs (SynaFlexer positioning device; Synarc Inc).^30^ Study inclusion was initially restricted to individuals with KLG 2 and 3 to ensure established OA and minimize ceiling effects. However, to enhance recruitment, a subsequent protocol amendment allowed inclusion of participants with KLG 1, who were then added to the KLG 2 cohort. Eligible individuals were required to have a summed pain score of 125 to 400 (greatest level of pain) on their more symptomatic (index) knee according to the Western Ontario and McMaster Universities Osteoarthritis Index (WOMAC), using a 0- to 500-point score visual analog scale, with the highest value indicating the most pain, stiffness, and limitation of function,^31,32^ and to be categorized within American Rheumatism Association functional class I (completely able to perform usual activities of daily living), II (able to do usual self-care and vocational activities but limited in avocational activities), or III (able to perform usual self-care activities but limited in vocational and avocational activities).^33^

Exclusion criteria included a concurrent medical or rheumatologic condition that could confound evaluation of the index joint, predominant patellofemoral disease (as determined by the investigator), a history of substantial trauma or surgery to the index knee, or a coexisting morbidity that jeopardized successful completion of the trial. The institutional review boards of both participating VA medical centers approved the study, and all patients gave written informed consent; financial compensation was provided. The trial protocol is available in Supplement 1. This study followed the Consolidated Standards of Reporting Trials (CONSORT) reporting guideline for randomized clinical trials.^34,35^

### Treatment Regimens

The study consisted of 3 distinct phases: period 1, initial screening; period 2, a 4-week prerandomization sham run-in period for the participants to develop facility in using the device; and period 3, a 48-week sham-controlled treatment period. Permuted-block randomization was used with random block sizes, stratified by clinical centers and baseline KLG. The randomization code list was developed by the Department of Veterans Affairs Cooperative Studies Program Coordinating Center in Hines, Illinois, using SAS, version 9.4 (SAS Institute Inc). Eligible patients were randomized (1:1) to daily self-administered 20-minute treatment with PLIUS or sham control. Based on the randomization, both sham and active devices were coded and distributed to participating research sites by the Department of Veterans Affairs Cooperative Studies Program Pharmacy Center.

Participants were allowed the use of acetaminophen (up to 3000 mg/d) and/or immediate-release tramadol (up to 200 mg/d) as rescue analgesia for severe knee pain throughout the trial, except for the 24 hours before each clinical evaluation. A stable daily dose of nonsteroidal anti-inflammatory drugs for pain unrelated to OA was also permitted. Patients were evaluated at baseline and 2, 4, 8, 12, 24, 36, and 48 weeks after randomization.

### Outcome Measures

The study was designed with symptom improvement and preservation of cartilage as coprimary outcome measures because each is considered an equally important target in the treatment of OA. Symptom reduction was examined using the Outcome Measures in Rheumatology Clinical Trials–Osteoarthritis Research Society International (OMERACT-OARSI) response rate.^36^ For this outcome, a clinical response is achieved if there is either greater than 50% improvement in pain and function with at least a 20% absolute improvement or at least 2 of 3 factors (pain, function, and patient global assessment) improve by at least 20%, with at least a 10-mm absolute improvement.

Disease progression in OA is typically accompanied by cartilage loss. Hence, the structural outcome measure used in this study was change in the thickness of the articular cartilage within the tibiofemoral joint from baseline to 48 weeks determined by magnetic resonance imaging (MRI) assessment of the central medial femoral condyle cartilage thickness.^37,38,39,40,41^ The MRI protocol developed for the Osteoarthritis Initiative on 3T MRI scanners (Siemens Healthineers) was adopted, because a large body of work exists validating this protocol for measurements of cartilage morphometry. Cartilage volumes were segmented manually after a first-pass automated segmentation using Colipe software version 14.10.1 revision 264 (Qmetrics Technologies). All final manual segmentations were performed by the same reader (M.D.T.), with blinding to time point in the study, with subcompartmental analyses of cartilage volume and thickness performed from which central medial femoral condyle cartilage thickness was derived. A difference of at least 33 μm between the PLIUS and sham group at 1 year was selected as a signal of positive response.

Secondary outcome measures, selected a priori in accordance with the preliminary recommendations of the OARSI task force,^42^ included pain, stiffness, and function WOMAC subscales along with the total score^32^; the patient’s global assessment of disease status^43^; response to therapy evaluated through the use of a 100-mm visual analog scale on which higher scores indicate more severe disease; the investigator’s global assessment of disease status and response to therapy, also assessed with a 100-mm visual analog scale; and constant, intermittent, and total pain subscales according to the Intermittent and Constant Osteoarthritis Pain scoring system, with higher values indicating more severe pain.^43^ Six serum and urine biomarkers of cartilage breakdown were also measured: serum C-terminal telopeptide of collagen II, urine C-terminal telopeptide of collagen II, serum cartilage oligomeric matrix proteins, serum collagen II cleavage, serum nitrated collagen II cleavage, and urine nitrated collagen II cleavage. Samples were collected at baseline and 24 and 48 weeks after initiation of the treatment period. Morning second-void urine samples were collected and frozen for urine biomarker measurements, with values adjusted using serum creatinine level.^44^ All biomarkers testing was conducted by Artialis Group.

### Adverse Events

Adverse events were assessed and graded for severity and attributability by the investigator (C.G.J. and K.H.) at the study visit. Adverse events included adverse device events, non–device-related adverse events, and serious adverse events. Adverse device events were further categorized into adverse reactions, suspected adverse reactions, and unanticipated adverse device events. An independent data and safety monitoring committee performed periodic reviews of adverse events.

### Device

PLIUS was self-administered using a lightweight, portable US Food and Drug Administration–approved device (Sonic Accelerated Fracture Healing System; Bioventus LLC), currently in clinical use for fracture healing.^45^ An investigational device exemption was obtained to use the device in this trial. The device produced a spatial average–temporal average power of 30 mW/cm^2^, with a sinusoidal waveform of frequency 1.5 MHz. The pulse burst frequency was 1 kHz and the duration was 200 ms. Adherence was ascertained through interrogation of the PLIUS device on repeat visits, with the number and duration of treatment sessions recorded internally.

### Analytic Plan

The purpose of this phase 2A trial was to determine whether potential superiority existed in either or both primary outcomes, which would justify a larger, more definitive study of PLIUS. For that reason, the sample size was calculated with a signal detection approach (ranking and selection method). No multiplicity adjustment for the 2 coprimary outcomes was conducted.^46,47,48^

The 48-week outcomes were assessed independently. For the 48-week OMERACT-OARSI response, the minimally clinically significant difference is an absolute rate difference of 10% between 2 groups. A total sample size of 144 provides a probability of at least 0.885 of detecting a treatment effect. For central medial femoral condyle cartilage thickness, a difference in measurement of 33 μm at 48 weeks, with a total sample size of 144, results in a 90% probability of detecting a positive effect.^37^ All other outcomes were secondary and considered exploratory.

### Statistical Analysis

Statistical analyses were conducted under intention-to-treat principles. SAS, version 9.4, was used for all analyses, and all comparisons were 2-sided with a significance level of *P* < .05. Comparison of baseline characteristics between PLIUS and sham groups was conducted using the *t* test, χ^2^ test, Fisher exact test, or Wilcoxon rank-sum test. Each coprimary outcome (ie, 48-week OMERACT-OARSI response and 48-week central medial femoral condyle cartilage thickness change score) was assessed independently and compared in the PLIUS and sham groups to determine whether the difference between groups exceeded the a priori–specified threshold. All outcome measures were analyzed using a mixed-effects model that included all available follow-up time points without adjustment for missing values. The key analysis for secondary outcomes was the interaction between time and treatment group.

## Results

### Participant Characteristics

**Figure 1.**
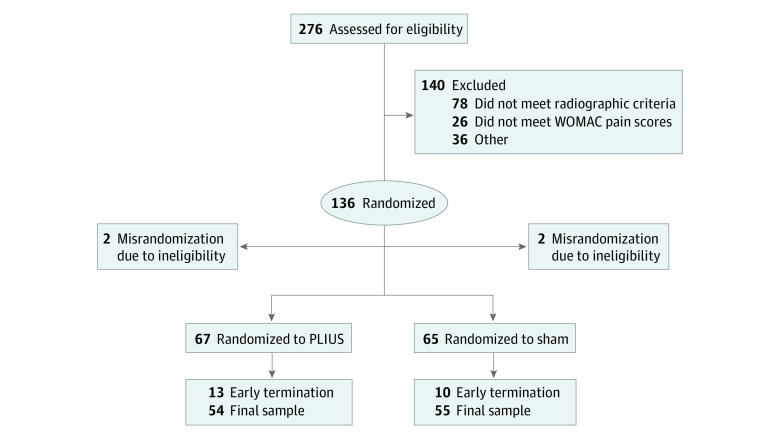
Participant Flow Diagram Outcome Measures in Rheumatology Clinical Trials–Osteoarthritis Research Society International (OMERACT-OARSI) was used as the symptomatic outcome measure in 54 patients in the pulsed low-intensity ultrasonography (PLIUS) cohort and 55 patients in the sham cohort. Central medial femoral condyle cartilage thickness was used as the structural outcome measure in 51 patients in the PLIUS cohort and 48 patients in the sham cohort. WOMAC indicates Western Ontario and McMaster Universities Osteoarthritis Index.

As detailed in Figure 1, a total of 4879 patients were screened and 276 patients were assessed for eligibility. There were 140 screen exclusions, leaving 136 randomized participants. The most common reasons for screen exclusion were not meeting radiographic criteria (78 patients [56%]) and WOMAC pain scores less than 100 or greater than 400 (26 [19%]). Among patients randomized, 4 were misrandomized and excluded as early terminations, leading to a total study population of 132. In addition, there were 27 terminations before completion of the study: 15 of 67 (22%) participants in the PLIUS group and 12 of 65 (19%) in the sham group.

Baseline characteristics are detailed in Table 1. The cohort self-identified as mean (SD) age, 63.6 (10.7) years, comprising 119 men (90.2%) and 13 women (9.8%). Self-reported race and ethnicity was as follows: American Indian/Alaskan Native, 8 (6.1%), Asian, 8 (6.1%), Black/African American (12 (9.1%), Native Hawaiian/Pacific Islander, 4 (3.0%), White, 108 (81.8%), and other (Latin, Mexican, Filipina, Spanish, and Puerto Rican), 7 (5.3%). The mean (SD) body mass index (calculated as weight in kilograms divided by height in meters squared) for 130 randomized participants was 31.7 (5.5). The mean (SD) duration of OA symptoms was 13.4 (12.3) years. There were 15 patients with KLG grade 1, 51 with grade 2, and 66 with grade 3. Other patient characteristics were well balanced between treatment groups. Ultrasonographic device adherence data averaged 90% or higher for both groups.

**Table 1.** Baseline Characteristics by Study Group

Characteristic	No. (%)		
	PLIUS (n = 67)	Sham (n = 65)	Total (N = 132)
Age, mean (SD), y	62.9 (10.5)	64.4 (10.9)	63.6 (10.7)
Sex			
Female	6 (9.0)	7 (10.8)	13 (9.8)
Male	61 (91.0)	58 (89.2)	119 (90.2)
Race^a^			
American Indian/Alaskan Native	5 (7.5)	3 (4.6)	8 (6.1)
Asian	5 (7.5)	3 (4.6)	8 (6.1)
Black/African American	7 (10.4)	5 (7.7)	12 (9.1)
Native Hawaiian/Pacific Islander	3 (4.5)	1 (1.5)	4 (3.0)
White	53 (79.1)	55 (84.6)	108 (81.8)
Other (Latin, Mexican, Filipina, Spanish, Puerto Rican)	5 (7.5)	2 (3.1)	7 (5.3)
Ethnicity			
Cuban	1 (1.5)	0	1 (0.8)
Mexican, Mexican American, Chicano	7 (10.4)	4 (6.2)	11 (8.3)
Not Spanish, Hispanic, Latino	54 (80.6)	55 (84.6)	109 (82.6)
Puerto Rican	2 (3.0)	2 (3.1)	4 (3.0)
Other (Spanish, Hispanic, Latino)	3 (4.5)	4 (6.2)	7 (5.3)
BMI, No.	66	64	130
Mean (SD)	31.8 (5.4)	31.6 (5.5)	31.7 (5.5)
Years with OA, mean (SD)	15.0 (13.2)	11.7 (11.2)	13.4 (12.3)
Years since OA diagnosis, mean (SD)	10.4 (10.6)	7.7 (9.8)	9.1 (10.3)
ARA functional class No. (%)^b^			
I	2 (3.0)	0	2 (1.5)
II	52 (77.6)	48 (73.8)	100 (75.8)
III	13 (19.4)	17 (26.2)	30 (22.7)
IV	0	0	0
Kellgren-Lawrence grade^c^			
1	8 (11.9)	7 (10.8)	15 (11.4)
2	25 (37.3)	26 (40.0)	51 (38.6)
3	34 (50.7)	32 (49.2)	66 (50.0)
Global assessment of disease status score, mean (SD)^d^			
Patient	50.4 (22.5)	53.0 (17.4)	51.7 (20.2)
Physician	58.0 (27.5)	63.7 (22.3)	60.8 (25.1)
WOMAC score, mean (SD)^e^			
Pain subscale	228.5 (76.0)	250.4 (78.5)	239.3 (77.7)
Stiffness subscale	97.9 (41.9)	109.0 (46.0)	103.4 (44.2)
Functional subscale	755.0 (316.7)	855.9 (276.0)	804.7 (307.1)
Total	1081.4 (407.6)	1215.3 (378.0)	1147.3 (397.5)

Abbreviations: ARA, American Rheumatism Association; BMI, body mass index (calculated as weight in kilograms divided by height in meters squared); OA, osteoarthritis; PLIUS, pulsed low-intensity ultrasonography; WOMAC, Western Ontario and McMaster Universities Osteoarthritis Index.

^a^ Race was reported by participant and more than 1 answer was allowed; hence, the percentages sum to more than 100.

^b^ Class I: completely able to perform usual activities of daily living, II: able to do usual self-care and vocational activities but limited in avocational activities, and III: able to perform usual self-care activities but limited in vocational and avocational activities.

^c^ Grade 1: doubtful narrowing of joint space, possible osteophytic lipping; 2: definite osteophytes and possible narrowing of joint space; and 3: moderate multiple osteophytes, definite narrowing of joint space, some sclerosis, and possible deformity.

^d^ Measured on a visual analog scale (0-100) rated by patient or clinician for overall assessment of knee arthritis severity. Larger numbers are more severe conditions.

^e^ Higher values indicate more pain, stiffness, and functional limitations.

### Outcomes

**Figure 2.**
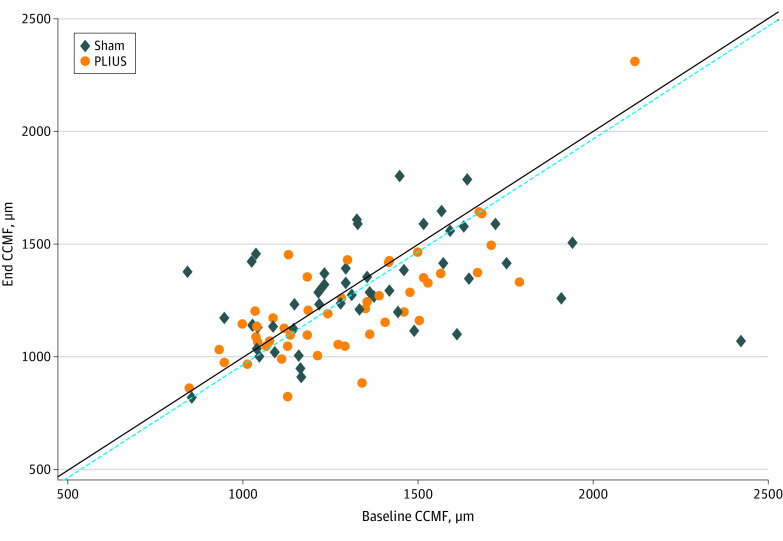
Baseline vs Final Cartilage Thickness Association between central medial femoral condyle cartilage (CCMF) before and after 48 weeks of pulsed low-intensity ultrasonography (PLIUS) or sham treatment. The solid line indicates no change. Data points above the dashed line represent structural responders, ie, loss in cartilage less than 33 μm.

The primary analysis of the symptomatic coprimary OMERACT-OARSI outcome showed a response of 70.4% (95% CI, 58.2%-82.6%) in the PLIUS group and 67.3% (95% CI, 54.9%-79.7%) in the sham group as assessed at 48 weeks of treatment (*P* = .84). The between-group difference of 3.1% (95% CI, –14.3% to 20.5%) did not meet the difference threshold of at least 10%. At week 48, the mean (SD) PLIUS group cartilage thickness decreased more than the sham group (73.8 [168.1] vs 42.2 [297.0] μm), with a between-group 48-week average change score of –31.7 μm (95% CI, –129.0 μm to 65.7 μm). This change did not meet the threshold of a cartilage difference of at least 33 μm. Most participants lost cartilage, with no statistically significant difference between the 2 groups (Figure 2).

Secondary outcome results are reported in Table 2. There were 53 serious adverse events and 188 postrandomization nonserious adverse events (99 in the PLIUS group, 89 in the sham group) during the trial. No serious adverse events were deemed related to the study device.

**Table 2.** Primary and Secondary Outcomes

Outcome	PLIUS			Sham			Difference	*P* value^a^
	No.	Mean (SD)	Change from baseline (95% CI)	No.	Mean (SD)	Change from baseline	Between PLIUS and sham (95% CI)	
**Primary outcomes**								
Cartilage thickness, μm								
Baseline	61	1321.1 (291.8)	–73.8 (–122.6 to –25.0)	61	1333.6 (334.1)	–42.2 (–126.6 to 42.2)	–31.7 (–129.0 to 65.7)	.44
48 wk	51	1257.5 (280.2)		52	1298.3 (216.0)			
OMERACT-OARSI, No. (%)								
Change	54	38 (70.4)	NA	55	37 (67.3)	NA	NA	.84^b^
**Secondary outcomes**								
WOMAC scale^c^								
Pain								
Baseline	67	228.5 (76.0)	–93.4 (–124.4 to –62.3)	65	250.4 (78.5)	–117.1 (–146.2 to –87.9)	23.7 (–18.4 to 65.8)	.55
48 wk	54	126.1 (121.0)		55	130.1 (116.5)			
Stiffness								
Baseline	67	97.9 (41.9)	–37.1 (–51.6 to –22.6)	65	109.0 (46.0)	–49.8 (–64.6 to –35.1)	12.7 (–7.7 to 33.2)	.67
48 wk	54	57.4 (51.7)		55	61.0 (54.3)			
Functional limitation								
Baseline	67	755.0 (316.7)	–283.1 (–399.7 to –166.5)	65	855.9 (290.3)	–407.3 (–510.8 to –303.7)	124.1 (–29.8 to 278.1)	.17
48 wk	54	447.6 (415.6)		55	450.9 (393.5)			
Total								
Baseline	67	1081.4 (407.6)	–413.6 (–571.4 to –255.7)	65	1215.3 (378.0)	–574.1 (–713.8 to –434.4)	160.6 (–47.61 to 368.8)	.24
48 wk	54	631.1 (581.8)		55	642.1 (554.6)			
Global assessment^d^								
Patient								
Baseline	67	50.4 (22.5)	–17.6 (–26.3 to –9.0)	65	53.0 (17.4)	–23.7 (–30.8 to –16.5)	6.0 (–5.1 to 17.1)	.84
48 wk	54	30.5 (26.3)		55	29.7 (25.9)			
Investigator								
Baseline	67	58.0 (27.5)	–18.9 (–27.9 to –10.0)	65	63.7 (22.3)	–28.6 (–36.6 to –20.6)	9.7 (–2.1 to 21.5)	.15
48 wk	54	38.6 (28.0)		55	35.1 (29.7)			
ICOAP subscales^e^								
Constant pain								
Baseline	67	42.5 (19.5)	–13.7 (–20.7 to –6.7)	65	44.7 (16.9)	–18.4 (–24.0 to –12.8)	4.7 (–4.2 to 13.5)	.92
48 wk	54	26.0 (24.8)		55	26.4 (22.4)			
Intermittent pain								
Baseline	67	47.3 (18.2)	–15.0 (–21.5 to –8.5)	65	51.1 (16.2)	–20.2 (–26.8 to –13.6)	5.3 (–3.9 to 14.4)	.91
48 wk	54	30.1 (23.7)		55	30.9 (21.2)			
Total pain								
Baseline	67	45.1 (17.6)	–14.4 (–20.8 to –8.0)	65	48.2 (14.7)	–19.4 (–25.2 to –13.6)	5.0 (–3.6 to 13.5)	.91
48 wk	54	28.2 (23.2)		55	28.8 (21.1)			
Medial JSW, mm								
Baseline	58	2.6 (1.2)	–0.0 (–0.1 to 0.1)	52	2.6 (1.1)	–0.1 (–0.2 to 0.0)	0.0 (–0.1 to 0.2)	.61
48 wk	51	2.6 (1.2)		48	2.5 (1.1)			
Lateral JSW, mm								
Baseline	58	4.7 (1.4)	–0.2 (–0.3 to 0.0)	52	4.8 (1.3)	0.0 (–0.1 to 0.1)	–0.2 (–0.4 to 0.0)	.06
48 wk	51	4.6 (1.5)		48	4.7 (1.2)			
Serum CTX-II, ng/mL								
Baseline	66	1.4 (0.5)	–0.1 (–0.3 to 0.0)	62	1.5 (0.6)	–0.2 (–0.3 to –0.1)	0.0 (–0.1 to 0.2)	.92
48 wk	55	1.3 (0.5)		54	1.4 (0.5)			
Urine CTX-II, ng/mL								
Baseline	66	305.5 (208.3)	37.8 (–20.3 to 95.9)	62	300.1 (235.0)	–11.6 (–78.3 to 55.2)	49.4 (–37.9 to 136.7)	.47
48 wk	55	333.0 (266.8)		54	309.1 (213.8)			
Serum COMP, ng/mL								
Baseline	66	1164.4 (647.9)	10.0 (–66.5 to 86.6)	62	1078.5 (367.3)	68.6 (–55.4 to 192.6)	–58.6 (–202.0 to 84.9)	.57
48 wk	55	1222.3 (748.4)		54	1149.0 (555.7)			
Serum Coll2-1, pg/mL								
Baseline	65	483.5 (157.5)	79.6 (34.2 to 124.9)	62	505.0 (179.3)	81.8 (45.3 to 118.3)	–2.2 (–59.7 to 55.3)	.80
48 wk	55	561.8 (159.5)		54	567.4 (233.4)			
Serum Coll2-1 NO2, pg/mL								
Baseline	66	337.2 (158.8)	112.3 (48.6 to 176.1)	62	360.6 (269.0)	136.2 (46.0 to 226.4)	–23.8 (–132.1 to 84.4)	.31
48 wk	55	463.5 (298.3)		54	494.7 (485.1)			
Urine Coll2-1 NO2/creatinine normalized, nM								
Baseline	66	40.6 (26.1)	–14.4 (–20.8 to –8.0)	62	41.8 (21.7)	–19.4 (–25.2 to –13.6)	5.0 (–3.6 to 13.5)	.63
48 wk	54	41.4 (23.1)		54	40.1 (23.5)			

Abbreviations: Coll2-1, collagen II cleavage; Coll2-1 NO2, nitrated collagen II cleavage; COMP, cartilage oligomeric matrix protein; CTX-II, C-terminal telopeptide of collagen II; ICOAP, intermittent and constant pain; JSW, joint space width; NA, not applicable; OMERACT-OARSI, Outcome Measures in Rheumatology Clinical Trials–Osteoarthritis Research Society International; PLIUS, pulsed low-intensity ultrasonography; WOMAC, Western Ontario and McMaster Universities Osteoarthritis Index.

^a^ *P* value determined from mixed-effects random intercept model, except where noted.

^b^ *P* value determined from χ^2^ test.

^c^ Higher values indicate more pain, stiffness, and functional limitations.

^d^ Measured on a visual analog scale (0-100) rated by patient or clinician for overall assessment of knee arthritis severity. Larger numbers indicate more severe conditions.

^e^ Higher values indicate more severe conditions.

## Discussion

Despite its substantial morbidity, there are no disease-modifying therapies approved for knee OA by the US Food and Drug Administration. In 2021, 2 review articles highlighted the lack of effective interventions for disabling symptoms other than total joint replacement.^3,8^ These facts provided the motivation for our trial of PLIUS in OA, together with promising in vitro and preclinical animal results.^19,23^ We implemented therapy with a US Food and Drug Administration–approved ultrasonography device for fracture healing for which there is moderate- to high-quality evidence of efficacy as defined by reduced time to fracture union and improved quality of life.^49^ The selection of coprimary outcomes (OMERACT-OARSI and central medial femoral condyle cartilage thickness) for the present trial reflects the need for a clinically meaningful advance in OA treatment to both mitigate symptoms and lessen cartilage degeneration.

In the present trial, neither coprimary outcome met its predefined threshold for benefit from PLIUS in treatment of knee OA. Symptomatic improvement occurred quickly in both the PLIUS and sham groups and was sustained for the duration of the study (eFigure in Supplement 2); the percentage of OMERACT-OARSI responders was 70.4% in the PLIUS group and 67.3% in the sham group, consistent with other therapeutic trials that have used this outcome.^50^ Both groups experienced cartilage loss (range, 42.2-73.8 μm) at 48 weeks, similar to the previously reported annual loss of 47 μm.^37^

Numerous secondary outcome measures were evaluated, including analgesia use as a potential signal of therapeutic response (eTable 1 and eTable 2 in Supplement 2). Consistent with the coprimary outcome measures, no significant differences between treatment and sham groups were observed for any secondary outcome measure.

The WOMAC score has been used extensively to quantify the symptoms of knee OA since its introduction in 1988.^31,32^ Both the sham and PLIUS arms exhibited rapid and sustained improvements in the WOMAC pain, stiffness, and function subscales, which has been seen in other OA trials,^50,51,52^ and no significant differences between the 2 study arms were seen at any time in the trial. Increased WOMAC pain scores have been associated with increasing KLG in other OA populations,^53^ but an increase was not observed in our study cohort. The large placebo effect associated with both patient-reported and physician-reported subjective measures in randomized clinical trials of OA has been considered and presents a major limitation.^54,55^

Use of MRI in evaluation of cartilage in OA remains a rapidly evolving field, with the ability of MRI methods to accurately assess cartilage morphologic factors continuing to improve. Since the inception of this trial, there have been major advances in 3-dimensional morphological MRI of knee cartilage. It is likely that newer techniques, with scan times equivalent to those used in the present work, would yield lower measurement error in the assessment of the central medial femoral condyle cartilage thickness. Alternatively, a rapid imaging protocol with comparable image quality to that in the present study^56^ has been adopted in several ongoing clinical trials.^57^ New ultrahigh-resolution (0.3-mm isotropic) 3-dimensional morphological imaging techniques at ultrahigh field strength (7 T) are also showing promise for rapid measurement of cartilage morphometry. However, based on the large SD of our cartilage thickness measurements, we recommend that the emphasis in future studies be placed on accuracy and precision, rather than measurement speed. In any event, these continuing advances in MRI methods have the potential to quantify OA therapeutics in a manner that is both sensitive and cost-effective.

We monitored serial serum and urine biomarkers of OA to evaluate the efficacy and time course of therapeutic response to PLIUS.^44,58,59^ Despite the wide range of biomarkers studied, no biochemical signal of therapeutic response was observed. This includes lack of a treatment effect in urinary C-terminal telopeptide of collagen II, which has been proposed as a predictor of radiographic change.^58^ We did not incorporate markers of synovial or bone response or of inflammation.^58^ Given the null results, no determination of biomarker sensitivity or positive predictive value was possible.^51^ Although changes from baseline to 48 weeks were seen in most outcomes, there were no statistically significant differences between groups for any of the outcomes consistent with the primary outcome results.

### Limitations

This study has limitations. Among the study’s limitations were barriers to recruitment, including primarily failure to meet KLG and WOMAC pain scores for entry. The study participants were drawn from a population receiving care through the VA, and as expected, most were men. Thus, the results may not be generalizable to the overall OA population. Subgroup analysis of men and women was not performed. We are not aware of sex differences reported in other studies of PLIUS. Accordingly, we believe that it is unlikely that results would differ in a female population. Furthermore, although adherence to device administration was excellent according to the criteria monitored by the device itself (>90%), adequacy of device placement could not be assessed. In addition, many challenges in the quantification of OA symptoms remain, including the placebo effects discussed. Although the placebo effects can be addressed through use of double-blind, placebo-controlled randomized clinical trial methods, ceiling and floor effects, as well as a much-narrowed dynamic range of outcomes, remain as substantial problems. In addition, our application of PLIUS was based primarily on its successful use in fracture healing. However, it is clear that optimal ultrasonographic parameters for cartilage repair may vary substantially from those that are effective for fracture healing.^60,61,62^ For example, our dosage was approximately 1% to 5% of that used by Loyola-Sánchez et al.^27^ We believe that this topic merits further study, given the documented anabolic effects of PLIUS for cartilage matrix in vitro and in preclinical animal studies and the current lack of any disease-modifying therapeutics for OA.

## Conclusions

Although PLIUS benefit was not observed and promising signals for future investigation were not immediately apparent, this clinical trial provides subjective, objective (joint space width by radiographic and cartilage thickening by MRI), and biomarker information. This information may aid future trial design by better defining expected ranges of values and rates of change. No signal was identified in this 48-week trial to suggest that PLIUS, applied as described, provides benefit for either OA symptoms or cartilage loss in knee OA.

## Notes

Supplement 1.
Trial Protocol

Supplement 2. **eTable 1.** Average Analgesia by Group **eTable 2.** NSAID Use During the Trial **eFigure.** Mean WOMAC Pain Subscale Over Time by Treatment Group

Supplement 3.
Data Sharing Statement
