# Supplementary material for: Effect of Pulsed Low-Intensity Ultrasonography on Symptom Relief and Tibiofemoral Articular Cartilage Thickness Among Veterans Affairs Enrollees With Knee Osteoarthritis: A Randomized Clinical Trial
Source: JAMA Netw Open. 2022 Mar 8;5(3):e220632. doi: 10.1001/jamanetworkopen.2022.0632 (PMC8905392; doi:10.1001/jamanetworkopen.2022.0632)
Supplement: Supplement 1. — Trial Protocol [file jamanetwopen-e220632-s001.pdf]

A Pilot Trial to Assess Low-intensity Ultrasound in Osteoarthritis

Cooperative Clinical Trial Award #0008

PROTOCOL

PRIVILEGED AND CONFIDENTIAL-NOT TO BE DISSEMINATED BEYOND OFFICIAL USE

June 1, 2017

Version 4.0

Table of Contents

I. SPECIFIC AIMS 4
I.A Objectives 4
I.B Specific Aims: 4
I.C Expected Outcome and Impact: 6

II. RESEARCH PLAN 6
II.A Background and Significance 6
II.A.1 Overview 6
II.A.2 Effects of PLIUS on Cartilage Repair 7
II.A.3 Challenges and Innovations in Clinical Trials in OA 8
II.A.4 The Osteoarthritis Initiative (OAI) and the Development of MRI Measures, Including Cartilage Thickness, as Sensitive Modalities for OA Assessment 9
II.A.5 Impact on Patient Care of Disease Modification in OA 10
II.A.6 Implications of Potential Study Outcomes 11
II.B Preliminary Studies 11
II.B.1 PLIUS in OA 11
II.B.2 MRI as a Measure for Cartilage Health 16
II.B.3 Experience of the Investigative Team 17
II.C Research Design and Methods 18
II.C.1 Overview of Study Design 18
II.C.2 Participant Recruitment & Retention 19
II.C.3 Schedule of Assessments 19
II.C.4 28-Day Screening Period 1 21
II.C.5 Inclusion and Exclusion Criteria 22
II.C.6 4-Week Prerandomization Sham Run-in Period 2 25
II.C.7 48-Week Sham controlled Treatment Period 3 27
II.C.8 Post End of Treatment Period 4 Safety Follow-up 29
II.C.9 Interim Visits 30
II.C.10 Compensation of Study Participants 30
II.C.11 Outcome Measures 31
II.D Biostatistical Considerations 35
II.D.1 Primary Outcome Measures 35
II.D.2 Hypothesized Treatment Effects 35
II.D.3 Ranking and Selection Procedures 36
II.D.4 Sample Size Calculation 36
II.E Statistical Analysis Plan 38
II.E.1 Baseline Characteristics 38
II.E.2 Analyses of Co-Primary Outcomes 38
II.E.3 Analyses of Secondary Outcome Measures and Secondary Analyses of Primary Outcome Measures 39
II.F Stratified Analyses 40
II.G Site Performance Monitoring 40
II.H Data Collection and Quality Control Measures 41

II.I MRI Acquisition and Quality Control Measures **42**
II.J Bilateral PA View Fixed-Flexion Knee Radiograph Acquisition and Quality Control Measures **43**
II.K General Guideline for Administration of Study Participant Questionnaires **44**
II.L Administration of the WOMAC Questionnaire **44**
II.M Exogen Device Quality Assurance **44**
II.N Study Organizational Structure **45**

III. Human Subjects **45**
III.A Risk to Participants **45**
III.A.1 Human Participant Involvement and Characteristics 45
III.A.2 Potential Risk 46
III.A.3 Sources of Materials 48
III.A.4 Therapeutic Risks 48
III.A.5 Research Risk 48
III.B Adequacy of Protection Risk **49**
III.B.1 Recruitment and informed consent 49
III.B.2 Protection Against Risk 50
III.C Potential Benefits of Research to Participants **52**
III.D Importance of Knowledge to be Gained **53**
III.E Data Monitoring and Reporting **53**
III.E.1 Data Monitoring 53
III.E.2 Data Reporting 54
III.F Safety Monitoring and Reporting **54**
III.F.1 Safety Monitoring 54
III.F.2 AE, SAE, ADE, and UADE Monitoring and Reporting Requirements 57
III.F.3 Reporting of Events of Special Concerns 58
III.G Data Monitoring Committee **58**
III.G.1 Reporting AEs, UADEs and SAEs to the Data Monitoring Committee (DMC) 58
III.H Females and Pregnancy Testing **58**
III.I Women, Minorities and Children **59**

IV. REFERENCES **60**

V. APPENDICES (not embedded within protocol) **67**
V.A CCTA 0008 MR Imaging Protocol **67**
V.B CCTA 0008 X-Ray Imaging Protocol **67**
V.C Knee Examination Protocol **67**

I. SPECIFIC AIMS

I.A Objectives

The objective of this Phase IIa clinical trial is to determine whether pulsed low-intensity ultrasound (PLIUS) may provide a therapeutic benefit for idiopathic knee osteoarthritis (OA). If so, this would strongly motivate a subsequent Phase IIb or III trial. The trial incorporates two co-primary outcomes: symptomatic improvement, assessed by OMERACT-OARSI Responder Criteria[11], and disease-modification, assessed by MRI-determined cartilage thickness.

Knee OA is a leading cause of age-associated disability with still greater prevalence in people with military service [12]; it is the most common indication for knee arthroplasty in the US. Therapies for pain are only modestly effective, and disease-modifying interventions for the cartilage degeneration accompanying OA are entirely lacking [13].

The proposed intervention is based upon the established fact that mechanical stimulation increases chondrocyte matrix production [14, 15]. PLIUS is a clinically feasible, easily-applied form of mechanical stimulation with longstanding use in orthopedics for fracture healing [16, 17]. The anabolic effects of PLIUS on chondrocytes have been established in basic science studies of cellular systems [18-24], cartilage explants [25, 26] and engineered cartilage constructs [27]. These anabolic effects have also been consistently observed in preclinical studies [5, 6, 28-30], including and perhaps most importantly, in the marked attenuation of cartilage degeneration in the Hartley guinea pig model of human OA [5].

Based on this substantial body of literature, we propose a Phase IIa trial of PLIUS as a potential therapeutic modality for knee OA in humans. PLIUS will be applied using a light and portable FDA-approved device, the Sonic Accelerated Fracture Healing System (SAFHS) [31] currently in clinical use for fracture healing. We will file an Investigational Device Exemption (IDE) for approval to use the SAFHS in this trial. This is a truly translational bench-to-bedside proposal targeting a very debilitating and highly prevalent disease with no disease-modifying and only marginally effective symptom-modifying therapies currently available. If this intervention is successful and subsequently confirmed in a pivotal trial, it would radically change the standard of care for this highly prevalent and debilitating disease.

I.B Specific Aims:

<u>Primary Hypothesis:</u>

1. PLIUS is potentially more effective than sham treatment as a disease- and symptom-modifying intervention for OA of the knee.

Aim 1A. Measure the change in cartilage thickness of the central, weight bearing, region of the medial femoral condyle after 48 weeks of PLIUS treatment compared to 48 weeks of sham-treatment in OA patients using MRI morphologic measures [32]. The 48-week time point is selected due to the expected time course of cartilage regeneration.

Aim 1B. In the same individuals, assess symptomatic response using the OMERACT-OARSI Responder Criteria [11]. OMERACT-OARSI is widely-used to assess response to OA treatment; response will be evaluated throughout the study. Note that the above hypothesis and associated aims form the basis of this Phase IIa clinical trial.

Secondary Hypotheses:

2. PLIUS is more effective than sham in treating knee pain and improving knee function associated with OA.

Aim 2. Assess the change in knee pain and function using the corresponding validated WOMAC scales [33] after 48 weeks of PLIUS treatment compared to sham-treatment in OA patients.

3. PLIUS is more effective than sham in decreasing severity of bone marrow lesions (BMLs) associated with OA as measured by BML maximal size assessed with MRI.

Aim 3. MRI analysis of subchondral BMLs using images obtained as part of the morphologic study of cartilage thickness will be performed. Bone marrow lesions have been consistently found to be associated with OA pain.

4. PLIUS is more effective than sham in decreasing serum and urine biomarkers associated with OA progression.

Aim 4. Soluble OA biomarkers will be collected concurrent with knee MRIs to evaluate the correlation between these outcomes. In addition, the change in these biomarkers at 48 weeks will be compared between treatment groups.

5. PLIUS is more effective than sham as a disease-modifying intervention for structural progression of knee OA as assessed by radiographic joint space narrowing (JSN).

Aim 5. JSN in the medial compartment of the treated knee will be assessed on a Fixed-Flexion (FF) radiograph [8] after 48 weeks of PLIUS treatment compared to sham. Radiographic JSN remains the FDA gold standard for disease modification.

Note that the above Secondary Hypotheses and associated Aims are purely exploratory in nature, and while they may guide further studies, they will not be incorporated as primary outcomes in the present study.

I.C Expected Outcome and Impact:

As discussed, there is a great deal of mechanistic and basic science support for the proposed intervention PLIUS. In addition, Loyola-Sanchez et al. have shown the feasibility of applying PLIUS to OA patients [34]. Further, OA is increasingly prevalent [35], particularly in veterans [12], and lacks both effective long-term symptom-modifying therapies and any disease-modifying intervention [13]. Demonstration of the hypothesized effects would strongly motivate a pivotal trial that could establish a major advance in OA treatment.

II. RESEARCH PLAN

II.A Background and Significance

II.A.1 Overview

Osteoarthritis (OA) affects almost 27 million Americans with an estimated net cost of over $80 billion/year [36]. In addition, the prevalence of OA is increasing rapidly, and it is estimated that by 2030 some 60 million Americans will be affected [37]. Further, OA is the leading cause of lower extremity disability in older age [35] and is the leading indication for total knee replacement (TKR). The number of TKR procedures has more than doubled over the last decade. OA is known to occur disproportionally in members of the armed services [12]. Within the VA system, 3351 TKRs were performed in FY02, while in the year ending March 31, 2012, 6698 TKRs were performed. Aside from phakectomy, TKR is the most common elective surgical procedure within the VA system. The immensity of the burden of OA is due largely to the fact that available medical management addresses only symptoms [13] and is of marginal long-term efficacy at best. In spite of significant progress in understanding the pathogenesis of OA, no disease-modifying interventions have been established. A number of surgical techniques to repair or replace damaged cartilage have been attempted, such as microfracture [38], autograft and allograft transplants [39], and autologous chondrocyte implantation [40] with disappointing long-term outcomes. Use of tissue engineered replacement cartilage is another approach that has generated a great deal of research interest, but to date has failed to yield any actual therapeutic products.

Degenerative articular cartilage is thought to be central to the pathogenesis of OA. However, articular cartilage has limited capacity for repair [41], and so the development of new cartilage regeneration and repair strategies is of great importance. Therefore, based on a large body of preclinical work demonstrating the anabolic effect of mechanical stimulation on chondrocyte metabolism, we propose to investigate the potential of PLIUS as a symptom and structure-modifying intervention in human subjects with early OA.

II.A.2 Effects of PLIUS on Cartilage Repair

Cyclic mechanical stimulation increases cartilage matrix production under a wide variety of circumstances [14], which is analogous to the anabolic effects of loading on bone. Ultrasound is a particularly convenient mode of delivering mechanical stimulation to human subjects. A large number of studies demonstrated the anabolic effects of PLIUS on bone, which led to the development of the Sonic Accelerated Fracture Healing System (SAFHS; Exogen®) introduced into clinical orthopedic practice in the United States for promoting the healing of certain fractures [42]. In addition to clinical results, there is a growing body of investigational literature on the mechanism by which ultrasound may exert its effects. Up-regulation of cycoloxygenase-2 via integrin signaling is thought to play an important role [43], while other proposed mechanisms include the exquisite sensitivity of certain enzymes (MMP1, collagenase) involved in cartilage homeostasis to the imperceptible degree of tissue heating induced by PLIUS [42].

A number of studies have demonstrated an ultrasound-induced increase in aggrecan gene expression and chondroitin sulfate synthesis for cartilage. Systems studied include chondrocytes harvested from embryonic chick sterna [18] and from the rabbit [19] and rat [20]; the healing fracture callus in the rat [21]; cells harvested from bovine [22]; rabbit [23] intervertebral disc; chondrocytes obtained from human OA patients [24]; and cultured explants from embryonic chick sterna [26] and from human joints [25]. Increased production of the two major macromolecular components of cartilage, proteoglycans and type II collagen, have been found in several of these ultrasound-stimulated systems [18, 22, 24-26]. It is important to emphasize that the energy of ultrasound used in these studies, as with the fracture healing device discussed above, is considerably lower than that used in physical therapy applications. In further work of particular relevance to tissue regeneration in situ, the up-regulation of appropriate matrix molecules in response to PLIUS was demonstrated in engineered cartilage over a time course [44]. This represents application of mechanical input as a therapeutic modality to an actual model of repair.

The mechanism underlying the increased matrix production by chondrocytes exposed to ultrasound is of substantial interest but remains unclear. Recent studies indicate that integrin signaling may play a central role in cartilage metabolism [45]. One report has indicated a difference in mechanotransduction between normal and OA chondrocytes and attributed that difference to altered integrin signaling [46]. Transduction pathways acting directly through integrin signaling may therefore represent major mediators of the effect of ultrasound on cartilage metabolism.

Ultrasound has also been applied to animal models of degenerative cartilage lesions. In two studies [28, 47] knee cartilage degeneration was induced in the rat by injection of papain. It was found that subsequent cartilage repair was

enhanced by ultrasound exposure in cases of mild pathology, while progression of the disease was attenuated. In another study, an acute chemical arthritis was induced in the radiocarpal joint of calves by intraarticular injections of turpentine oil [29]. Again, the effect of ultrasound applied over the joint was to improve the histologic state of the cartilage. Salutary effects of ultrasound were similarly observed in donkeys [48]. It was found that application of ultrasound to surgically induced full-thickness osteochondral lesions in the rabbit knee resulted in a statistically significant improvement in joint pathology and in the histology of repair cartilage [6]. Ultrasound in conjunction with high-molecular weight hyaluronate was found to be effective in reducing OA pathology in a rabbit model [49]. Finally, ultrasound has been successfully implemented as a therapeutic modality for the prevention and attenuation of the OA disease process in the guinea pig [5]. This is of substantial importance since the Hartley guinea pig develops spontaneous age-associated OA and is a particularly good model for idiopathic OA in humans [50]. The evidence from these preclinical studies set the stage for the conduct of the proposed Phase IIa trial using a device that has already undergone Phase I testing for promoting bone healing.

II.A.3 Challenges and Innovations in Clinical Trials in OA

Clinical trials in OA are rendered particularly problematic by the relatively subjective nature of pain and function as outcome measures used to assess efficacy, varied manifestations from patient to patient, and insensitivity and lack of specificity of knee radiographs which are used to assess structural progression. OA clinical trials generally address the dual objectives of improvement in OA pain and functional impairment. Generally these trials are complicated by high rates of placebo response. One method of addressing this is to implement a flare design in which the patient is withdrawn from background medication, experiences the expected increase in pain, and is then assessed over a relatively short time (2-6 weeks) for improvement in pain following application of an agent or placebo. Several studies have evaluated pain and function over longer periods of time or without flare design and have generally shown higher placebo response rates and less dramatic improvement in pain and function.

An advance of potentially great significance would be the development of an intervention that alters OA disease progression through attenuating the progression of anatomic findings seen with worsening disease. This is entirely analogous to the case of rheumatoid arthritis, in which the development of disease-modifying agents has revolutionized treatment. For OA, evaluation of disease-modifying agents is greatly complicated by the use of plain radiographs as outcome measures. The primary radiographic outcome of joint space narrowing (JSN) is an indirect and relatively insensitive marker of articular cartilage status, and in addition depicts only a two-dimensional representation of the gross bony changes associated with late-stage OA. There is increasing evidence that for knee OA, direct assessment of cartilage thickness in three dimensions by MRI is a more precise structural outcome measure[51] than radiography. In

addition, bone marrow lesions (BMLs) can be identified on MRI and are associated with articular pain in OA [52]. This proposed trial will evaluate symptoms and structure using current standard outcome measures for both pain and function, and structural improvement. In addition, outcomes will include important state-of-the-art quantitative measurements of cartilage thickness in a targeted region, and of the maximal size of BMLs by MRI. BMLs in particular have been shown to be associated with both cartilage loss and knee pain [52, 53].

Finally, in terms of the practicality of the proposed treatment, Loyola-Sanchez and colleagues recently reported application of PLIUS in 27 patients with knee OA. Their study, while too small to detect a treatment response, directly demonstrated the feasibility of this therapeutic approach [34].

II.A.4 The Osteoarthritis Initiative (OAI) and the Development of MRI Measures, Including Cartilage Thickness, as Sensitive Modalities for OA Assessment

There has been considerable activity over the past decade in the development of robust imaging-based biomarkers of disease activity for OA. In particular, morphometric measurement of cartilage volume and thickness using magnetic resonance imaging (MRI) is increasingly used as a structural endpoint for progression of OA, [54, 55], [56]. Cartilage thickness in particular has been shown to be very sensitive to change and progression of OA, notwithstanding the fact that OA is a disease of the entire joint [57]. High-resolution 3D MRI techniques such as double-echo steady-state (DESS) imaging at 3 Tesla are well suited for quantitative assessment and monitoring disease progression of knee OA [58] and have been used for assessing longitudinal changes in the OAI-and the OAI ancillary-Pivotal OAI MRI Analyses (POMA: HHSN26820100002, PI: Kwoh CK)[59].

The OAI (http://oai.ucsf.edu/) is a nationwide multi-center longitudinal cohort study sponsored by the National Institutes of Health. Launched in 2004, the overall objective of the OAI is to establish a public database resource to aid in the identification of biological and behavioral biomarkers, including MRI, for the development or progression of knee OA for use in epidemiologic studies and to evaluate therapeutic strategies in clinical trials. (http://oai.epi-ucsf.org/datarelease/docs/StudyDesignProtocol.pdf). The OAI collects extensive annual clinical, radiographic and MRI data on a cohort of 4,796 men and women ages 45 to 79 with a planned eight years of follow-up. MRI protocols of the OAI include high-resolution (3T) proton sagittal DESS imaging with water-excitation sequences on both knees to assess joint and cartilage morphology. The OAI cohort is divided into subcohorts (i.e., ~28% progression subcohort, ~68% incidence subcohort, and ~4% reference subcohort). MRI data are also available for a subset of the progression cohort at 18 and 30 months. These images form the basis for many studies of MRI volume and thickness measurements, and define the sensitivity of cartilage thickness as a biomarker of OA progression. Many of the recent studies extend the

analysis to femorotibial subregions in the cartilage, using sophisticated computer-based segmentation tools, [60], [56, 61]. Dr. C. Kent Kwoh, who is PI of one of the NIH funded OAI Clinical Centers and a consultant on the present study, will provide an important link between the OAI protocols and the study design, data collection, retention strategies and image acquisition processes that we will use.

In addition to cartilage morphometric measurements, the OAI MRI dataset permits whole-joint evaluation according to several semi-quantitative OA scales that combine features relevant to functional integrity of the knee. These features include cartilage morphology, subchondral BMLs and cysts, osteophytes, meniscal and ligamentous integrity, synovial properties, joint effusion, bone attrition, intra-articular loose bodies, and periarticular cysts or bursitis [62]. These semi-quantitative knee scores include the Whole-Organ MRI Score (WORMS) [63, 64]. Several variations and improvements on WORMS have been proposed, including the Boston-Leeds OA Knee Score (BLOKS) [64], and most recently the MRI Osteoarthritis Knee Score (MOAKS) [62].

Accurate and repeatable measurement of cartilage thickness requires consistency and careful control of both MR image acquisition and subsequent segmentation and analysis. Despite these challenges, cartilage thickness by MRI is emerging as a robust indicator of disease modification in OA. The OAI has established a detailed and highly repeatable protocol for image acquisition, and other studies [60, 61] have quantified the efficacy of tracking OA progression using cartilage thickness measurement from OAI data. Our group of investigators includes experts with extensive experience in quantitative MRI of articular cartilage and experienced statisticians. The MRI acquisition protocol and analysis approach proposed in this study are the result of careful analysis of the OAI data and published analyses. Our MRI acquisition mirrors that used on the 4,796 subjects in the OAI, and we are adopting best available practices in image segmentation and analysis.

II.A.5 Impact on Patient Care of Disease Modification in OA

OA is the most common rheumatologic condition. Its incidence is increasing with the aging of the population and with the epidemic of obesity. It is particularly prevalent in individuals with military service [12]; by age 75 the vast majority of veterans will have symptomatic OA. Current therapies are only marginally effective and are aimed exclusively at managing symptoms. In addition, the analgesics and anti-inflammatory drugs used for OA are frequently not tolerated by the target population, which is comprised disproportionately of individuals of advanced age and with common comorbidities such as hypertension, diabetes mellitus and renal insufficiency. Along with these limitations on medications that relieve OA symptoms, there are no FDA-approved interventions for OA that alter disease progression. Indeed, OA is the single most common indication for total joint replacement. If data from this Phase IIa study suggest

that PLIUS is effective in treating OA symptoms and has the potential to ameliorate disease progression, it would strongly motivate a definitive follow-up trial. If confirmatory in a follow-up pivotal trial, this would then have a major impact on clinical management of OA. If disease modification in particular is established, PLIUS could greatly impact the now spiraling costs of surgical OA management in the US [65] and within the VA. This burden will continue to increase along with the increasing prevalence of OA.

II.A.6 **Implications of Potential Study Outcomes**

As detailed above, knee OA is a highly prevalent disease with only modestly effective long-term medical options for symptomatic therapy and no FDA-approved structure-modifying interventions. Note that while PLIUS is an extremely promising therapy, there are at present insufficient data on the application of this modality to human OA patients to justify a full, pivotal, clinical trial. We are instead seeking a signal of efficacy for symptomatic improvement and for disease modification. Thus, there are four possible outcomes of the proposed preliminary trial, depending on the signal found for these two primary endpoints: 1) If our results suggest that PLIUS may be effective both for symptom relief and to ameliorate disease progression, it would strongly motivate a subsequent definitive clinical trial to further substantiate these effects; 2) If the results suggest that PLIUS is effective for symptoms but not for structure modification, a definitive trial to more clearly establish and quantify the extent of symptomatic improvement and further affirm safety issues would be indicated. This would help to determine the role of PLIUS in the symptomatic management of OA; 3) Results suggesting that PLIUS has efficacy in structure modification but not for symptomatic improvement would suggest that OA pain and functional limitations on the one hand, and structural progression on the other, may not be linked pathophysiologically. This would suggest a potential role for PLIUS in disease prevention or prophylaxis, for example, following knee injury, to preserve cartilage health. A thoughtfully designed follow-up study would therefore be of substantial interest; and 4) Results demonstrating no improvement in symptoms or structure would suggest that, in spite of substantial basic science evidence, there may be no PLIUS effect in human OA or that the selected dose, duration or mode of administration may need to be reconsidered.

II.B **Preliminary Studies**

II.B.1 **PLIUS in OA**

II.B.1.a **Anabolic Effects of Ultrasound on Chondrocytes and Cartilage**

We have applied ultrasound to chondrocytes [18], cartilage explants [26], engineered cartilage tissue constructs [44], and OA in an animal model [5]. In all cases, the application of ultrasound was through use of the sonic accelerated

fracture healing system (SAFHS), an FDA-approved device in clinical use for fracture healing (Exogen®). This is the same device that we will use in this Phase IIa trial of PLIUS in human subjects.

Chondrocyte study [18]: PLIUS at an FDA-approved level for fracture healing, 30 mW/cm^2, and at 2 mW/cm^2, was applied to chondrocytes in culture in a single application. The effect on cell proliferation and on expression of mRNA for collagen II, collagen X, Sox9 (a gene associated with collagen II synthesis), and aggrecan was followed for a period of one week in addition to immunostaining for collagen II and aggrecan. Overall anabolic effects of PLIUS were documented, with the lower power resulting in effects that were at least as large as those seen with the higher power. This is in agreement with literature results indicating that the ultrasound effect may require a small threshold signal, rather than following a usual dose-response profile.

Cartilage explant study [26]: PLIUS was applied to chick sternal explants. Quantitative immunohistochemical staining was used to evaluate regional production of collagen II, collagen X (as a marker for chondrocyte hypertrophy, indicating a pre-ossification state), and aggrecan. The proximal and distal portions were investigated separately, due to their different developmental fates (ossifying and non-ossifying, respectively). Immunohistochemistry demonstrated increased aggrecan and collagen II (below). Type X collagen was not produced in the distal part of the sternum, indicating that ultrasound did not induce a tendency towards ossification in that region.

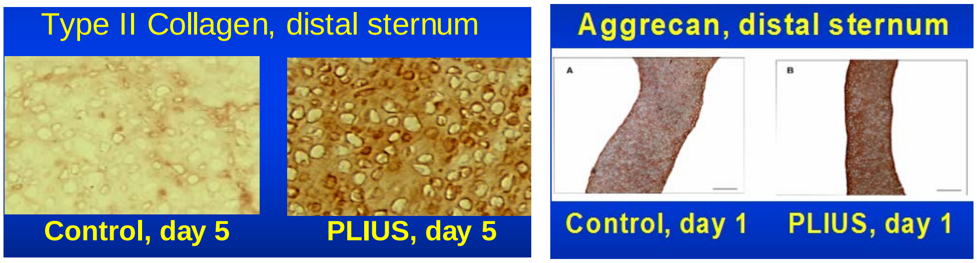

Immunohistochemical staining of control and PLIUS-treated chick sternal explants. The increase in production of type II collagen and aggrecan in the distal, non-ossifying, part of the sternum is illustrated.

Tissue regeneration study [44]: We tested the hypothesis that cartilage regeneration would be augmented by application of PLIUS, and that matrix-sensitive MR parameters could be used for non-invasive assessment of this phenomenon. As a model, we used bovine stifle joint chondrocytes and a type I collagen scaffold, which has been shown to support the chondrocyte phenotype. In order to interpret the response seen in the standard MR outcome measures T_1, T_2, and k_m, we performed correlative histologic, immunohistochemical, biochemical, and FT-IR measurements. Biochemical data showed increased PG at 3 and 5 weeks of growth in PLIUS-treated as compared to untreated constructs (left hand panel below), and increased collagen at 3 weeks (right hand panel below). A decrease in total collagen at 5 weeks reflects breakdown of the biodegradable type I collagen scaffold.

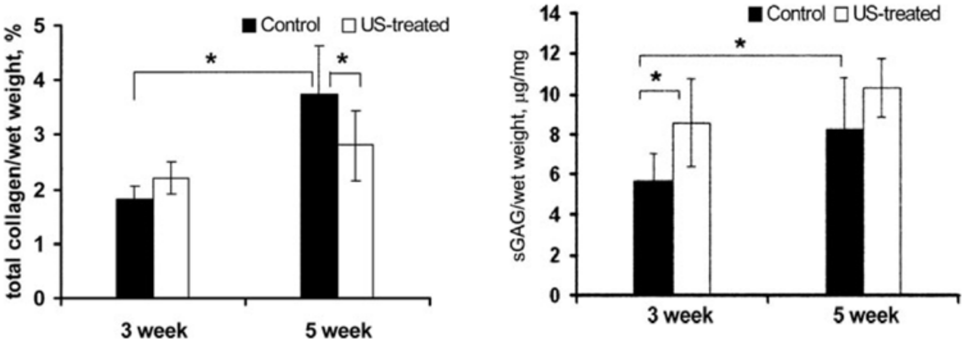

Effect of PLIUS application to tissue engineered cartilage constructs (see text), showing the resulting increase in matrix production. The apparent loss of collagen at 5 weeks is due to collagen scaffold breakdown.

Immunohistochemical results indicated more precisely the composition of the construct. As shown in the panel below, overall staining for collagen types I and X was weak, as was staining for MMP-13. Collagen type X and MMP-13 staining intensities were further decreased with PLIUS. In contrast, collagen type II staining was strong for all groups, and increased with time in culture. More intense pericellular collagen type II staining was seen in the 5-week PLIUS-treated group, with significant augmentation by PLIUS at that time point.

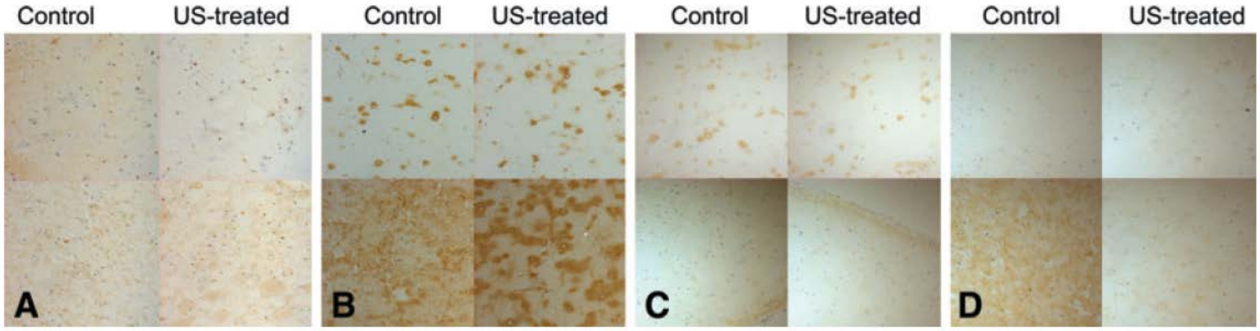

Immunostaining results for 3 weeks (upper row) and 5 weeks (lower row) of tissue development under PLIUS as compared to control constructs. A: Collagen I; B: Collagen II; C: Collagen X; D: MMP-13.

Corresponding results were obtained using Fourier transform infrared spectroscopic analysis, evaluating the absorbances at wave numbers specific to particular macromolecular components. Finally, MRI experiments indicated consistency with the above interpretation of increasing macromolecular content, and hence decreased molecular mobility, as reflected by decreased T_2 and increased k_m; again, the decrease in k_m and trend to increase in T_2 at 5 weeks is due to the breakdown of the collagen scaffold.

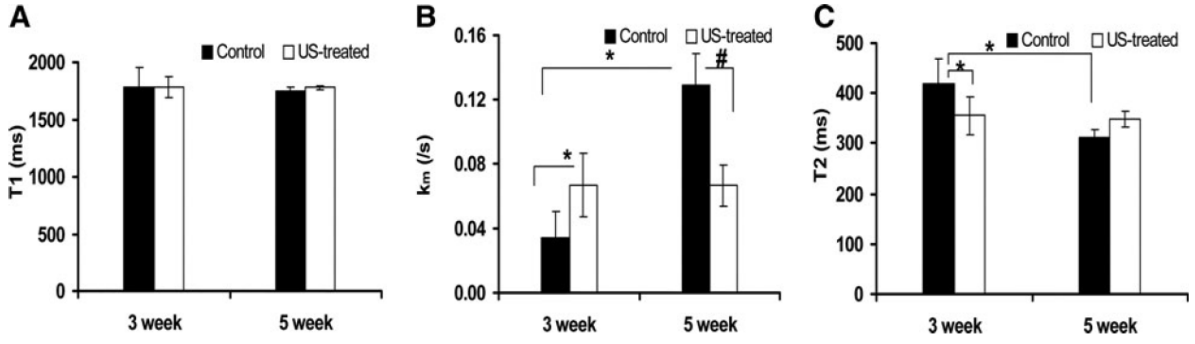

MRI analysis of the effect of PLIUS treatment on (A) T1, (B) km, and (C) T2 measurements of collagen constructs (n=10 per group). Although there were no significant changes in T1 with PLIUS, km increased while T2 decreased at 3 weeks, reflecting matrix maturation. The decreases at 5 weeks are due to scaffold breakdown. *p<0.05 versus 3-week control, #p<0.05 versus 5-week control.

Together, these results strongly support our hypothesis that PLIUS induced anabolic effects in engineered cartilage constructs, and that these effects could be monitored noninvasively by MRI.

Animal model study [5]: The Hartley Guinea pig develops spontaneous age-associated OA and is a particularly good model for idiopathic OA in humans [50]. We used this to test the efficacy of PLIUS in the treatment of OA. Four groups were studied: i) treatment initiated at two months of age, treatment duration of four months (E2/T4); ii) treatment initiated at two months of age, duration ten months (E2/T10); iii) treatment initiated at twelve months of age, duration three months (E12/T3); iv) treatment initiated at twelve months of age, duration six months (E12/T6). Groups i) and ii) test efficacy in preventing pathology, while groups iii) and iv) provide results related to retarding progression of, or reversing, established pathology. Treatment was applied to one limb, with the contralateral limb serving as a control. Overall, the cartilage from the treated limbs exhibited less staining loss and greater uniformity, less fibrillation, and less deep fissuring than did cartilage from control limbs. These effects were seen in all joint compartments. Representative histology is shown below.

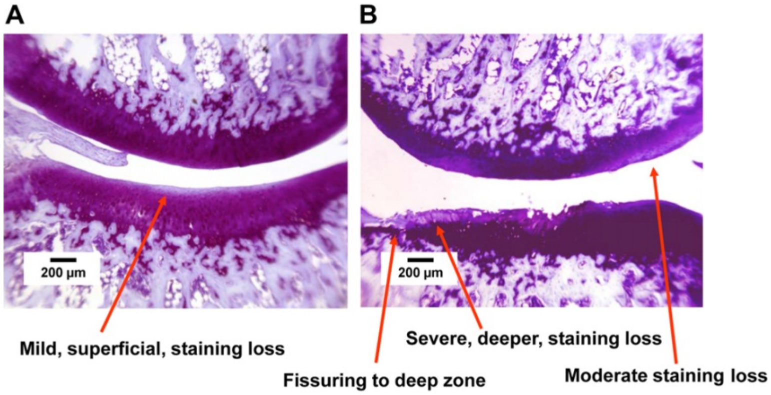

Illustrative results for treatment of animals initiated at 2 months of age, with treatment duration of 4 months. Left: PLIUS-treated. The medial tibial plateau (MTP) shows an intact surface with mild loss of matrix staining. The medial femoral condyle (MFC) shows an intact surface with uniform matrix staining. Right: Untreated control animal. The MTP shows extensive surface fibrillation and deeper fissuring, with greater loss of staining. Staining is with thionin.

Quantification of these effects was performed using a modified Mankin scale [66] assessing fibrillation (1 - 5), matrix distribution (1 - 4), chondrocyte loss (1 - 3), and chondrocyte cloning (1 - 4), with a minimum (best) score of 4 and a maximum (worst) score of 16. Scoring results are shown in the table below. Note that the intention of this study was to evaluate treatment at earlier as compared to later stages of disease. Therefore, we compared disease treatment initiated at 2 months of age with treatment initiated at 12 months of age. We were secondarily interested in treatment duration, and therefore divided each of these two groups into two treatment duration arms. While data were presented for all subgroups in the original publication, the prevention and treatment groups each in fact consisted of two treatment durations, as shown in the Table below:

Modified Mankin scores for tibial plateau

	Medial tibial plateau		Lateral tibial plateau		Average tibial plateau	
	Right knee: control	Left knee: PLIUS	Right knee: control	Left knee: PLIUS	Right knee: control	Left knee: PLIUS
Groups E2/T4 + E2/T10	**9.12 ± 1.67**	**8.47 ± 1.28***	**7.44 ± 0.95**	**7.13 ± 0.78 &**	**8.28 ± 0.96**	**7.80 ± 0.80****
Groups E12/T3 + E12/T6	10.43 ± 1.40	10.15 ± 1.35	8.12 ± 0.66	7.93 ± 1.00	9.27 ± 0.85	9.04 ± 0.96

Modified Mankin scores for femoral condyle

	Medial femoral condyle		Lateral femoral condyle		Average femoral condyle	
	Right knee: control	Left knee: PLIUS	Right knee: control	Left knee: PLIUS	Right knee: control	Left knee: PLIUS
Groups E2/T4 + E2/T10	7.34 ± 1.00	7.17 ± 0.83	6.73 ± 0.90	6.89 ± 1.15	7.03 ± 0.86	7.03 ± 0.90
Groups E12/T3 + E12/T6	8.24 ± 1.39	7.89 ± 0.83	**7.16 ± 0.74**	**6.14 ± 1.22****	**7.70 ± 0.92**	**7.07 ± 0.77***

Statistical comparisons are between control and treated knees. Bold indicates statistically significant differences (**P< 0.01; *P< 0.05) or trends towards significant differences (&P < 0.10). In addition to favorable trends throughout nearly all outcomes, statistically significant results are especially pronounced in the tibial plateau for the prevention group. Overall, PLIUS demonstrated substantial efficacy.

Finally, immunostaining was performed on cartilage from animals in which PLIUS was initiated at eight weeks of age, for a duration of 4 months. Chondrocyte expression of MMP-3, MMP-13, IL-1ra, and TGF-β1 in the femoral condyles was

quantified using a Bioquant Image Analysis System. PLIUS had a striking effect on the expression level of TGF-β1, which was expressed by chondrocytes throughout the full extent of the articular cartilage of the femoral condyle. Panel A below shows a typical example of the widespread expression of TGF–β1 throughout the articular surface of a non-treated joint. The expression of TGF–β1 was often observed to be associated with chondrocytes found in colonies, which we interpret as part of a widespread repair response (Panel B). Panel C shows the widespread expression of TGF–β1 in a non-treated joint from another representative animal. In contrast, the expression of TGF–β1 was dramatically reduced in articular cartilage exposed to the PLIUS treatment (Panel D).

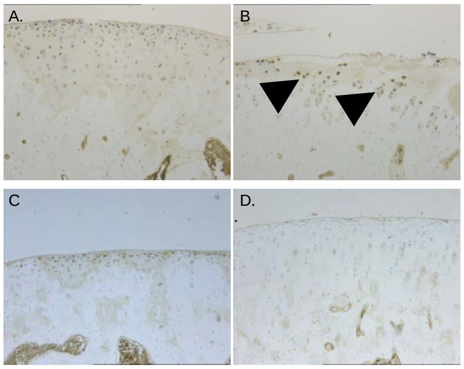

TGF-β1 expression. A) Femoral condyle from control knee joint. B) Higher magnification of joint surface shown in A. Arrowheads indicate enhanced expression of TGF-β1 in areas where chondrocyte proliferation, indicative of a repair response, is occurring. C) Femoral condyle from the non-ultrasound treated joint of another animal. D) The ultrasound–treated joint contralateral to the joint pictured in A.

II.B.2 MRI as a Measure for Cartilage Health

It is well-established that disease progression in OA is typically accompanied by structural changes in the joint, including loss of cartilage volume and thickness. In human studies, the standing anteroposterior (AP) knee radiograph has been the standard for measurement of joint space narrowing (JSN) for more than 30 years, but this technique is severely limited as a means to reproducibly visualize the thickness of articular cartilage [67] for a number of reasons. The intrinsically two-dimensional nature of the measurement requires particularly consistent joint positioning to obtain reproducible results. However, precisely reproducible positioning of the knee in serial examinations is extremely difficult; for example, differences in joint pain from examination to examination have been shown to affect positioning [68]. The Fixed-Flexion (FF) [8]views address some of these alignment issues and are superior to the standing AP view, but repeatability and accuracy still suffer from variations in positioning. Further difficulties arise from the fact that the x-ray measurement of cartilage thickness is based on hard tissue outlines, while cartilage itself is not visualized. Therefore, the status of other soft tissue structures, especially the menisci, affects the measurement of cartilage thickness.

Quantitative measurement of cartilage thickness from 3 Tesla MRI is emerging as an excellent and highly stable alternative to radiographic measurements [69]. Furthermore, data from more than 4700 subjects in the OAI has

established a clear relation between cartilage thickness changes as assessed by MRI and radiographic disease status [70]. A recent study by Wirth et al. [56] compared the standardized response mean (SRM), a measure of the sensitivity to change, and annualized rates of change in femorotibial cartilage plates and subregions for a 2-year vs. a 1-year study period using a subset of the progression cohort of the OAI. Significant structural progression was observed even over a 1-year observation period. In the central portion of the central medial femoral condyle, Wirth et al. measured baseline to 1-year mean change of -2.5% and an SRM of -0.31.

II.B.3 Experience of the Investigative Team

Inception, design, execution and reporting of federally funded controlled clinical trials in rheumatic diseases have been the career-long academic focus of the PI, Dr. Clegg. These have included trials with the Cooperative Systematic Studies in Rheumatic Diseases (CSSRD) early in his career. Later, with Cooperative Studies Program Coordinating Center (CSPCC) at the Hines VA, he was study chairman of CSP 341, Comparison of Sulfasalazine and Placebo in Spondyloarthritis [71-74]. Dr. Clegg was principal investigator (PI) for the NIH funded Glucosamine/chondroitin Arthritis Intervention Trial (GAIT) that addressed the efficacy of glucosamine, chondroitin sulfate or the two in combination in OA [75]. This included an investigator initiated exploratory study to evaluate structural progression [76, 77]. Dr. Spencer has longstanding interest in cartilage biochemistry and biophysics, with over 120 peer-reviewed publications in this and related areas. As part of this, he has investigated the anabolic effect of pulsed low-intensity ultrasound (PLIUS) on isolated chondrocytes, cartilage explants, tissue engineered cartilage constructs, and in the guinea pig model of idiopathic osteoarthritis. The proposed clinical trial is a direct outgrowth of these studies. In addition, Dr. Spencer has published extensively on MR imaging analysis of cartilage status. Dr. Spencer is currently co-investigator on one NIH-funded R01 grant and one R21 grant related to musculoskeletal MRI. The Hines CSPCC has nearly 40 years of experience supporting the planning, conduct and analysis of multi-center randomized clinical trials. Dr. Reda is the Center Director and has been involved in more than 30 multi-center randomized clinical trials, including GAIT and CSP 341. Kimberly Carlson, the lead biostatistician for this proposal, has performed statistical analyses for studies in several disease areas, including Parkinson's disease, diabetes management and metabolic syndrome. Dr. Kwoh, currently Division Chief at the University of Arizona Department of Medicine, is a consultant and a member of the Executive Committee for the study. He is also the PI on two NIH contracts for the University of Pittsburgh Clinical Center for the Osteoarthritis Initiative (OAI) (N01 AR 1-2262), which includes a research-dedicated OAI 3 Tesla imaging facility, and the Pivotal OAI MRI Analyses (POMA) (HHS N26820100002). Dr. Kwoh is also the Director of the University of Pittsburgh Multidisciplinary Clinical Research Center for rheumatic and musculoskeletal diseases (UPITT-MCRC) (P60AR054731). The overall objective of POMA is to identify imaging biomarkers of knee OA development and progression using quantitative and semi-quantitative MRI analyses. As a result of the OAI and POMA studies, he has extensive experience with the recruitment,

enrollment, and retention of participants with knee OA as well as the acquisition and analysis of 3 Tesla MRI images for quantitative cartilage thickness and volume measurement [78, 79]. A total of 1173 of the 4796 OAI participants were enrolled at the University of Pittsburgh and over 90% were still enrolled as of the 84-month visit. The knee pain and function and MRI outcome measures in the proposed study are closely based on those used in the OAI protocol. Neal K. Bangerter, PhD is an expert in musculoskeletal MRI, MR pulse sequences, and MR image analysis. He is currently a co-investigator on an NIH-funded R01 "MRI for Early Detection of Osteoarthritis", and was co-investigator on an NIH R01 "Rapid MRI for Evaluation of Osteoarthritis" (completed 2008) as well as on a GlaxoSmithKline sponsored project "Sodium MRI of articular cartilage at 3.0T for Drug Discovery" (completed 2007). Dr. Bangerter has published more than 25 journal articles and peer-reviewed proceedings related to MRI of the musculoskeletal system. Dr. Erika Schneider has been retained as a consultant for the study. Dr. Schneider is an expert in the operational setup and execution of multi-site, multi-vendor imaging-based biomarker studies. She is also a consultant to the OAI and was responsible for providing oversight of the OAI MR protocol, quality assurance (OA), image analysis, and the OAI CRO. Taken together the team assembled has a wealth of experience in the development of innovative controlled clinical trials in rheumatology; osteoarthritis; PLIUS application to cartilage; and advanced MR imaging analysis of OA and cartilage biology. We merge the strengths of the Cooperative Studies Program and the intramural efforts of the National Institute on Aging in a novel approach to a common but very difficult to treat disease with no FDA-approved structure-modifying therapeutic interventions available.

II.C **Research Design and Methods**

II.C.1 **Overview of Study Design**

This is a Phase IIa, multi-center, randomized, sham-controlled, parallel, double-blind trial with a prerandomization four-week sham run-in period to determine if PLIUS is potentially more effective than sham as a disease and symptom modifying intervention in patients with early OA of the knee. It is recognized that this proposed trial is shorter in duration than the current standard of 2 years for Phase III trials based on radiographic outcomes. Our objective at this early stage is to determine whether there is sufficient evidence of efficacy to warrant further investigation in a Phase IIb or III trial. If a signal were detected, it would also provide data to calculate the sample size required for the next trial. The study consists of four periods: a Screening Period 1, a 4-Week Prerandomization Sham Run-in Period 2, a 48-Week Sham-controlled Treatment Period 3, and a 30-day Post End of Treatment Safety Follow-up Period 4 for participants with unresolved Serious Adverse Event(s) (SAE) at study completion. A total of 180 patients will be randomized. All patients will be followed during the Treatment Period for 48 weeks, which is the time point for assessment of the primary outcome measures.

II.C.2 Participant Recruitment & Retention

Study participant recruitment will be the responsibility of the local principal investigator (PI) and the research staff. Potential study participants will be identified and recruited using a number of strategies. Research staff will educate their clinical colleagues at staff meetings regarding this study opportunity and will maintain frequent contact with them so potentially eligible patients seen during routine outpatient care visits will be referred to the study. Clinical staff, including rheumatologists, orthopedists, family practitioners, internists and musculoskeletal radiologists, will also be continually informed about the study through IRB approved print or web media, such as internal hospital communications (e.g., newsletters). IRB approved advertising (e.g., flyers or brochures) will be placed in clinic rooms and patient care areas as allowed by each center's policies.

As permitted by each study site, study participants with a diagnosis of OA of the knee will be identified through a search of patient databases. Study participants identified through this search will have their medical history pre-screened by study staff to determine potential eligibility per inclusion and exclusion criteria. Potentially eligible study participants will be told about this study by their primary or specialty care providers at upcoming appointments or by mail and instructed how to contact study site staff if interested in participating. Study participants expressing interest in participating after having an opportunity to learn more about the study will be pre-screened by phone or in person by study site personnel. If eligible based on the pre-screening information, they will be scheduled to come in for the informed consent process and the Screening visit.

II.C.3 Schedule of Assessments

The table on the page below contains the chronological sequence of the assessments and procedures to be performed:

PERIOD	NAME	Screening / Period 1	Sham Run-In / Period 2					Sham-controlled Treatment / Period 3													Post End of Treatment / Period 4
	**DURATION**	**Up to 4 weeks**	**4 Weeks**					**48 Weeks**													
VISIT	NUMBER	1	2	3	4	5		6	7	8 (6th)	9	10	11	12 (7th)	13 (8th)	14 (9th)	15 (10th)	16 (11th)	17 (12th)	18 (13th)	
	Change Note: V4 may be TC, window removed for V1-5.see new footnote 5. Visit 6, 7, 9, 10, 11no longer req'd. visit # in ( ) match visit # in ICF	**Screen**	**Begin Period 2**	**TC¹**	**Clinic⁵**	**Clinic (End of Period 2)**	**Begin Period 3**	**TC**	**Clinic**	**Clinic**	**TC**	**Clinic**	**TC**	**Clinic**	**TC**	**Clinic**	**TC**	**Clinic**	**TC**	**Clinic (End of Period 3)**	**Safety Follow-up²**
		**-28 Days to Day 0 Period 2**	**Day 0 Period 2**	**Wk 1 [+/-3d]**	**Wk 2 [+/-3d]**	**Wk 4 [+/-3d]**	**Day 0 Period 3**	**Wk 1 [+/-3d]**	**Wk 2 [+/-3d]**	**Wk 4 [+/-1wk]**	**Wk 6 [+/-1wk]**	**Wk 8 [+/-1wk]**	**Wk 10 [+/-1wk]**	**Wk 12 [+/-1wk]**	**Wk 18 [+/-1wk]**	**Wk 24 [+/-1wk]**	**Wk 30 [+/-1wk]**	**Wk 36 [+/-1wk]**	**Wk 42 [+/-1wk]**	**Wk 48 [+/-1wk]**	**30 days post Period 3 [+/-1wk]**
**Informed consent**		X						Visit removed in Protocol V4 and is not longer required	Visit removed in Protocol V4 and is not longer required		Visit removed in Protocol V4 and is not longer required	Visit removed in Protocol V4 and is not longer required	Visit removed in Protocol V4 and is not longer required								
**Inclusion/Exclusion Criteria Review**		X	Re-Confirm				Re-Confirm														
**Medical History Review**		X																			
**Physical Examination**		X																		X	
**Vital Signs (BP, HR, Temperature)**		X					X									X				X	
**Weight, Height³**		X					X			X				X		X		X		X	
**ARA Functional Class Assesment**		X																			
**Fixed Flexion Knee Radiograph**		X					X													X	
**WOMAC Osteoarthritis Index**		X	X				X			X				X		X		X		X	
**ICOAP Questionnaire**		X	X				X			X				X		X		X		X	
**Patient Global Assessment of Disease Status**		X	X				X			X				X		X		X		X	
**Investigator Global Assessment of Disease Status**			X				X			X				X		X		X		X	
**Patient & Investigator Global Assessment of Response to Therapy**										X				X		X		X		X	
**Investigator Knee Examination**		X	X				X			X				X		X		X		X	
**Dispense & Review of Rescue Medication Diary**		X	X		X		X			X				X		X		X		X	
**Review Analgesic Therapy Use**		X	X	X	X	X	X			X				X	X	X	X	X	X	X	
**MRI of Index Knee**							X									X				X	
**Urine Pregnacy Test⁴**		X					X														
**Adverse Events, Adverse Device Events & Serious Adverse Review**			X	X	X	X	X			X				X	X	X	X	X	X	X	X²
**OA Biomarkers**							X							X		X				X	
**Study Device Dispense / Return; Compliance Review**			X		X	X	X			X				X		X		X		X	
**Follow-up Telephone Call**				X											X		X		X		

¹ TC = Telephone Call; ² Post end of treatment safety follow-up by telephone for unresolved ADEs and SAEs; ³ Height performed at Visit 1 only;
⁴ Only for females of; childbearing potential; ⁵ V4 may be conducted by telephone if approved by site investigator.

V6.1 6/1/17

The length of study, the potentially delayed or slow rate of treatment response, daily frequency of treatment sessions, or visit schedule, to name a few, can potentially adversely affect study participant retention. The research staff will strive to minimize withdrawals from the study and will take measures to mitigate withdrawals by, for example, maintaining regular contact with study participants through clinic and telephone contact and encouraging study participants to voice any concerns or issues that arise as a result of their participation so that the research staff can discuss their concerns and as appropriate offer potential solutions that may prevent unnecessary withdrawals. While the research teams will strive to minimize withdrawals, study participants will not be coerced or intimidated to remain in the trial and will be advised that they always have the option to withdraw for any or no reason at all. However, if a patient elects to withdraw from treatment, they will be asked whether they would be willing to remain in the study for continued data collection as per the protocol schedule.

II.C.4 **Screening Period 1**

The Screening visit will occur prior to the Prerandomization Run-in Day 0 visit. At the Screening visit patients will undergo the following according to the schedule of assessments: Western Ontario and McMaster (WOMAC®) Index patient questionnaire; patient global evaluation of disease status; standardized physical examination of the knees; physical exam; Intermittent and Constant Osteoarthritis Pain (ICOAP) patient questionnaire; height, weight, vital signs (e.g., temperature, sitting blood pressure, sitting pulse rate & respiratory rate) and demographics; analgesic medication and other therapies for osteoarthritis use; urine pregnancy test for women of childbearing potential. Descriptions of questionnaires completed by patients can be found in SectionII.C.12.b. Sample WOMAC and ICOAP questionnaires can be found with the Case Report Forms. Assessment of Adverse Events (AEs), Adverse Device Events (ADEs) and Serious Adverse Events will begin after informed consent is obtained. The investigator and participant will decide which knee is the more symptomatic knee and designate that knee as the index joint. In the event that both knees are equally symptomatic, the right knee will be designated the index knee. Only the index knee will receive treatments with the study device.

At the Screening visit study participants will also have bilateral Fixed-Flexion knee radiographs [8]. From the Fixed-Flexion radiographs, K-L Grade and joint space width will be determined by the central reader to verify the diagnosis of OA of the knee and establish the potential for response. Radiograph acquisition and interpretation is discussed in Section II.J ..

Study participants will be allowed to take non-steroidal anti-inflammatory drugs (NSAIDs) , and will also be allowed to take acetaminophen (APAP) up to 3000mg/day and/or tramadol immediate release (IR) up to 200 mg/day as rescue analgesia for severe knee pain throughout the trial. Participants should be on a stable dose of NSAID for at least 7 days prior to visit 2 (Day 1 of run-in period) and avoid changes in dose and type of NSAID throughout the trial. The investigators selected APAP and tramadol as rescue medication because they would be the best tolerated, least toxic and least likely to cause problems that might lead to early withdrawal from the study due to adverse events. Rescue analgesics or NSAIDs will not be provided to the patients by the study. If not prescribed by the study participant's primary care physician, directions for use and appropriate prescriptions for NSAIDs and rescue medication in accordance with the protocol and standard of care will be provided by the site investigator. Study participants will be instructed to use as little rescue analgesia as possible by reserving its use for the relief of uncontrolled pain and will be given a diary to record date, name and amount of rescue medication used. It should take patients about five minutes per day to record daily rescue medication (i.e., acetaminophen, tramadol) in a diary. While reliability and data quality are often cited as drawbacks to diary use, they offer a systematic method of collecting data on rescue medication (RM) use and may also provide for a systematic assessment of a patient's perspective on symptoms and efficacy. To address reliability and data quality concerns, study staff will explain at the beginning and throughout the trial the importance of the RM diary, how it is to be used and, when appropriate, make attempts to correct deficiencies in RM diary completion and use. A similar diary was effectively used in GAIT [75]. Study participants must discontinue NSAIDs and rescue analgesia 24 hours prior to assessment of outcome measures and will be contacted prior to each study visit and reminded to avoid NSAID and analgesia use for 24 hours preceding each visit.

If at the Screening visit a study participant is taking an excluded concomitant treatment, it must be discontinued and the Run-in Day 0 visit should not occur until the applicable washout period(s) has been completed. A list of excluded therapies with their minimal washout periods will be provided to study sites Patients taking low-dose aspirin (325 mg/day or less) for vascular indications may continue to do so throughout the study. Patients taking excluded analgesics, including all narcotic analgesics, must discontinue use at least two weeks prior to the Run-in Day 0 visit.

II.C.5 Inclusion and Exclusion Criteria

<u>Inclusion Criteria</u>

Eligible patients must meet all of the following inclusion criteria:

1. Study participant gives voluntarily written informed consent to participate in the study prior to completion of any study-related procedures.

2. Male or female, at least 40 years of age, with a clinical diagnosis of primary OA of the index knee (tibiofemoral joint) based on clinical and radiographic criteria (Kellgren and Lawrence Grades 1, 2 or3) [80] as specified below at the Run-in Day 0 visit:
    - **i)** Pain in the index knee on motion or weight bearing for the majority of days during the previous month, at least partially relieved by rest; **AND**
    - **ii)** Kellgren and Lawrence Grades 1, 2 or 3 in the index knee as determined by the central radiology review.
3. Clinical symptoms of OA for at least 6 months prior to the Run-in Day 0 visit.
4. Sum of WOMAC pain subscale (Section A) between 125 and 400 mm inclusive in the index knee at the Day 0 **Period 3** visit.
5. American Rheumatism Association (ARA) Functional Class I, II, or III [81] at the Screening visit.

Class I	Completely able to perform usual activities of daily living (self-care, vocational, and avocational)
Class II	Able to perform usual self-care and vocational activities, but limited in avocational activities
Class III	Able to perform usual self-care activities, but limited in vocational and avocational activities
Class IV	Limited in ability to perform usual self-care, vocational, and avocational activities

6. Females of childbearing potential must be willing to use a reliable form of medically acceptable contraception (Examples of acceptable contraception include, but may not be limited to: oral/parenteral/implantable hormonal contraceptives, intrauterine device or barrier and spermicide). Women who are surgically sterile or have been post-menopausal for at least 2 years are not considered to be of child-bearing potential. Abstinence only is not an acceptable method.

Exclusion Criteria

Eligible patients must meet none of the following exclusion criteria:

1. Concurrent medical/arthritic disease that could confound or interfere with evaluation of pain or efficacy including: Inflammatory arthritis (e.g., rheumatoid arthritis, systemic lupus, spondyloarthropathy, psoriatic arthritis, polymyalgia rheumatica), gout, episodes of acute monarticular arthritis clinically consistent with pseudogout, Paget's disease affecting the index knee, a history of septic arthritis or avascular necrosis or intra-articular fracture of the index knee, Wilson's disease, hemochromatosis, alkaptonuria, or primary osteochondromatosis.
2. Spine or hip pain of sufficient magnitude to interfere with the evaluation of the index knee.
3. Isolated patellofemoral disease manifested by primarily anterior knee pain in the absence of tibiofemoral radiographic finding.
4. History of significant collateral ligament, anterior cruciate ligament or meniscal injury to the index joint requiring

surgical repair.

5. History of arthroscopy of the index knee within 6 months prior to the Run-in Day 0 visit.
6. Participant has a valgus deformity of greater than 15 degrees in the index knee per the investigator's knee examination or has a valgus deformity of greater than 10 degrees in the index knee as determined by the central radiographer.
7. History of any illness that in the opinion of the investigator might confound the results of the study or pose additional risk to the patient.
8. Female study participants who are breastfeeding, pregnant or plan to become pregnant during the study or within 3 months following the last study treatment.
9. Corticosteroid treatment as follows:
    a. Use of oral corticosteroids within the previous four weeks.
    b. Exposure to intramuscular corticosteroids within one month prior to the Run-in Day 0 visit.
    c. Administration of intra-articular steroids to the index knee, within 3 months of the Run-in Day 0 visit.
    d. Administration of intra-articular steroids to any other joint, within 1 month of the Run-in Day 0 visit.
10. Patients taking excluded therapy must discontinue use in accordance with the washout schedule before the Run-in Day 0 visit. Aspirin (up to 325 mg/day) for cardiovascular reasons may be continued. Excluded analgesics or aspirin for analgesic purposes, may be used during the trial for treatment of acute pain unrelated to knee OA, such as for treatment of pain related to a dental procedure, only if such use is no more than 14 consecutive days and is discontinued in accordance with the washout schedule prior to a clinic study visit.
11. Known contraindications to MRI scanning (e.g., pacemaker, metal fragments, spinal nerve stimulators and/or a body habitus that would preclude a patient from having an MRI).
12. Intra-articular injection of hyaluronic acid or congeners into the index knee within 6 months of the Run-in Day 0 visit.
13. Topical analgesics (e.g., capsaicin preparations) to the index knee or any oral analgesics (e.g., opiates containing analgesics) with the exception of permitted NSAIDs, acetaminophen and tramadol within 2 weeks of the Run-in Day 0 visit or during the study.
14. Implementation of any medical therapy, complementary or alternative regimens for the treatment of osteoarthritis within 7 days prior to the Run-in Day 0 visit.
15. Participation in another clinical study with an investigational agent within 4 weeks of the Run-in Day 0 visit.
16. Exposure to glucosamine or chondroitin sulfate within one month of the Run-in Day 0 visit.
17. Initiation of physical therapy or muscle conditioning program to the lower extremities within 2 months of the Run-in Day 0 visit.
18. Concurrent use of the following medications or interventions:
    a. Disease modifying antirheumatic medications including methotrexate, leflunomide, sulfasalazine and

hydroxychloroquine.

b. Chronic therapy with tetracycline or tetracycline derivatives.

19. Inability to understand and complete study questionnaires including questions requiring a visual analog scale (VAS) response.
20. Any condition that in the opinion of the investigator prevents adherence with the protocol or compliance to study treatment.

Study participants meeting entry criteria at screening will be eligible for the Run-in Day 0 visit.

II.C.6 4-Week Prerandomization Sham Run-in Period 2

It is anticipated that a 4-week Prerandomization Sham Run-in Period will enrich the patient population with those more likely to be compliant with the treatment regimen and increase the likelihood of demonstrating a positive result. The doxycycline in OA trial [10] successfully implemented a run-in period. In this trial's run-in period, 463 patients were enrolled and 32 patients failed to complete the run-in period (29 patients missed appointments, 3 patients had <80% medication compliance) yielding a 7% run-in period dropout rate. We project a slightly higher run-in period dropout rate of 10% for the proposed trial given that adherence to a daily treatment regimen involving a 20 minute treatment session is likely more demanding than taking oral medication. Therefore, we plan to enroll at least 200 patients in order to randomize a minimum of 180 patients.

The Run-in Period will consist of a follow-up telephone call, a follow-up clinic visit (if deemed appropriate by the site investigator this clinic visit may be conducted by telephone to reduce the burden for select participants who have significant difficulty traveling to clinic), and an End of Run-in clinic visit at weeks 1, 2 and 4 respectively after the Run-in Day 0 visit.

<u>Run-in Period 2 - Day 0 Visit</u>

The Run-in Day 0 visit is the beginning of the 4-week Prerandomization Sham Run-in Period and will occur after completion of the Screening visit. Prior to this visit, participants will be contacted and reminded to return their completed rescue medication diary and to discontinue NSAID and rescue analgesia 24 hours prior to their scheduled Day 0 Run-in visit. At this visit study participants will be re-evaluated to confirm that they meet eligibility criteria as previously described.NSAID andrescue medication use and diary will be reviewed, and participants will be reminded to use as little rescue analgesia as possible by reserving its use for the relief of uncontrolled pain. Study participants will also undergo all other assessments and procedures as detailed in the Schedule of Assessments.

Eligible patients will be enrolled in the Run-in Period and given a sham device. Patients will not know this is a sham device, however, as they will only be informed that all participants will receive sham device treatments during some portion of their participation in the study. Participants and research staff will be informed that there is no detectable difference between the sham and PLIUS devices. The devices appear and operate similarly, except the sham device does not generate an ultrasound signal. The PLIUS device does not generate vibration, warmth or other sensations that may un-blind research staff or participants. Patients will be shown how to properly operate and correctly position the device over the medial compartment of the index knee. They will then be asked to demonstrate proper treatment administration technique. Once the participant has verbalized and demonstrated understanding, they will be asked at this visit to self-administer their first 20-minute daily treatment. The Exogen device is intended for use in environments in which radio frequency interference (RFI) is controlled. At each visit addressing adherence to the treatment regimen, participants will be instructed on how to help prevent RFI with the Exogen device to ensure the device works at full capacity during treatments. Participants will receive a Participant Device Brochure for reference on the Exogen device, treatment administration process and preventing RFI.

Run-in Period 2 - **Week 1 Follow-up Telephone Call**

To ensure that study patients are not experiencing difficulty properly administering daily treatments and are compliant with the treatment regimen, patients will receive a follow-up telephone call at visit 3.0 approximately 1 week after the Run-in Day 0 visit. Adverse Events (AEs) and other analgesic therapy use will also be assessed at this visit.

Run-in Period 2 - Week 2 Follow-up Clinic Visit

Participants will have a follow-up clinic visit approximately 2 weeks from completion of the Run-in Day 0 visit. This visit may be conducted by telephone if deemed appropriate by the site investigator to reduce the burden for select participants who have significant difficulty traveling to clinic. Prior to presenting for this visit, study participants will be contacted and reminded to discontinue NSAID and rescue analgesia 24 hours prior to their scheduled visit, and to bring with them to the visit their rescue medication diary and study device. Treatment compliance data stored in the device will be reviewed and documented by the study staff. Participants will be withdrawn if they are less than 60% compliant [34] with the daily treatment regimen. NSAID use, rescue medication use and diary, other analgesic therapy use, and Adverse Events (AEs) will be reviewed. At this visit participants will be asked to review and demonstrate the treatment administration procedures they follow to administer a treatment using their assigned study device. Participants will not, however, actually turn on the device to administer a treatment. The importance of preventing RFI will also be reviewed. The study staff will observe their administration technique and make recommendations as appropriate to ensure proper

administration of study treatment. Participants will be reminded to use as little rescue analgesia as possible by reserving its use for the relief of uncontrolled pain.

Run-in Period 2 - Week 4 End of Run-in Clinic Visit

The Week 4 End of Run-in clinic visit (named in the Schedule of Assessments as the Week 4, end of Period 2 part of visit 5) marks the end of the Run-in Period and will occur approximately 4 weeks from completion of the Run-in Day 0 visit. Prior to presenting for this visit, participants will be contacted and reminded to discontinue NSAID and rescue analgesia 24 hours prior to their scheduled visit, and to bring with them to the visit their rescue medication diary and study device. Treatment compliance data stored in the device will be reviewed and documented by the study staff. Participants with less than 80% treatment compliance [34] will be withdrawn from the study. Participants who successfully complete the Run-in Period will then at this same clinic visit undergo baseline assessments and procedures per the Schedule of Assessments.

II.C.7 **48-Week Sham controlled Treatment Period 3**

II.C.7.a **Period 3 - Baseline Assessment**

Participants who successfully complete the Run-in Period as determined by assessments performed at the Week 4, End of period 2 part of visit 5, will undergo baseline assessments at the Day 0, Beginning of Period 3 part of visit 5 to confirm that they meet randomization criteria as previously described and are eligible to be randomized into the 48-Week Sham-controlled Treatment Period. Participants at baseline must have a summed score of 125-400 mm inclusive in Pain Section A of the WOMAC questionnaire to qualify for randomization into the study. The investigator will perform an examination of the index knee and global assessment of disease status. NSAID, other analgesic therapy and rescue medication use and diary will be reviewed, and biomarker samples will be collected. Adverse Events (AEs) will be assessed and recorded when applicable. A urine pregnancy test will be performed on all females of childbearing potential prior to randomization into the study. Study participants will also undergo all other assessments and procedures as detailed in the Schedule of Assessments and will be reminded to use as little rescue analgesia as possible by reserving its use for the relief of uncontrolled pain.

Study participants randomized into the study will have a baseline MRI to evaluate cartilage thickness of the medial condyle of the medial segment of the index knee [32, 56], and a bilateral PA view Fixed-Flexion [8] radiograph of the knees to establish the baseline radiographic status of the medial compartment of the knee. Knee MR and radiographic imaging protocols are discussed later in this proposal.

Eligible participants will be randomized to the PLIUS or sham device and as in the Run-in Period will apply the study device for 20 minutes daily over the medial compartment of the index knee. Participants will review and be expected to demonstrate proper treatment administration technique. The importance of preventing RFI will also be reviewed.

II.C.7.b **Period 3 - Randomization Methods**

The Hines CSPCC will develop the randomization scheme and associated codes. Permuted block randomization will be employed to assign patients to PLIUS or sham device. Randomization will be stratified by study site and Kellgren-Lawrence grade (KLG) 1, 2 and. 3.

Eligible patients will be randomly assigned to PLIUS or sham device using a web-based central randomization system with a telephone call randomization system as back-up. The Study Coordinator at the participating facility will be required to sign into this password-protected site. The Study Coordinator will enter the patient study number and will be asked to answer a few questions about eligibility in order to complete the randomization procedure. The website will then assign a randomization number to a study participant. The site will select the double-blind device with that randomization number and dispense the device to the study participant. A copy of appropriately executed and completed consent and Health Insurance Portability and Accountability Act (HIPAA) authorization documents must be on file at the Hines CSPCC, preferably before the patient is randomized or within 24 hours of randomization.

II.C.7.c **Period 3 – Follow-up Clinic Visits**

During the Treatment Period, study participants will have follow-up clinic visits scheduled from the day of randomization at Weeks 4, 12, 24, 36 and 48. Prior to presenting to the clinic for each follow-up clinic visit, study participants will be contacted and reminded to discontinue NSAID and rescue analgesia 24 hours prior to their scheduled visit, and to bring their rescue medication diary and study device to the visit. Patients will have an MRI of the index knee at Weeks 24 and 48. A Fixed-Flexion [8] radiograph of the knees will be performed at Week 48. Urine and blood samples will be collected at Weeks 12, 24 and 48 for OA biomarkers analyses. Treatment compliance data stored in the device will be reviewed and documented by the study staff. Study participants with less than 80% [34] or greater than 125% treatment compliance will be counseled as appropriate. Device surveillance will occur at each visit to ensure study devices are operating properly. At each follow-up clinic visit study participants will be asked to review and demonstrate the treatment administration procedures they follow to administer a treatment using their assigned study device. Participants will not, however, actually turn on the device to administer a treatment. The study staff will observe their administration technique and make recommendations as appropriate to ensure proper administration of study

treatment. The importance of preventing RFI will also be reviewed. During the Treatment Period study participants will also undergo all other assessments and procedures per the Schedule of Assessments including: WOMAC; patient and investigator global evaluations of disease status and response to study treatment; examination of the index knee; collection of urine and blood samples for biomarker analyses; ICOAP; weight and vital signs (temperature, sitting blood pressure, sitting pulse rate & respiratory rate will only be collected at Weeks 24 & 48); Adverse Events (AEs) assessment and collection when applicable; NSAID, other analgesic therapy and rescue medication use, and diary review. Study participants will also be reminded to use as little rescue analgesia as possible by reserving its use for the relief of uncontrolled pain.

II.C.7.d **Period 3 – Follow-up Telephone Calls**

To ensure study participants are not experiencing difficulty properly administering their daily treatments and are compliant with the treatment regimen and preventing RFI, follow-up telephone calls will be conducted at Weeks 18, 30 and 42, which are the midpoints between clinic visits at weeks 12, 24, 36 and 48. Adverse Events and use of NSAIDs and other analgesic therapies will also be assessed during each follow-up telephone call.

II.C.7.e **Period 3 – Additional Safety Follow-up**

Study participants will be monitored per the Schedule of Assessments at each visit for Adverse Events (AEs), which includes Adverse Device Events (ADEs) and Serious Adverse Events (SAEs) that occur after informed consent is obtained. Safety follow-up monitoring for unresolved SAEs and ADEs will occur every 30 days by phone or during a routine clinic visit, until resolved or resolved with sequelae and the participant is clinically stable.

II.C.7.f **End of Study/Early Termination**

The End of Study will occur when the last randomized study participant, has completed the Week 48 visit. In the event a study participant prematurely and permanently discontinues treatment (for any reason) during the Treatment Period 3 all assessments planned for the Week 48 visit per the Schedule of Assessments must be performed no later than 2 weeks following the date of the last administration of study device.

II.C.8 **Post End of Treatment Period 4 Safety Follow-up**

Participant's with unresolved Adverse Device Events (ADEs) and Serious Adverse Events (SAEs) at completion or early termination will be contacted by phone 30 days after their completion of, or early termination from, the study. This follow-up assessment will be documented in the appropriate study case report form. In addition, each investigator will record and report all ADEs and SAEs they are made aware of that occur within 30 days of a participant's completion of or

early termination from the study. Investigators will ensure that participants with unresolved ADEs and SAEs requiring follow-up care after study completion or early termination are referred for appropriate follow-up care.

II.C.9 **Interim Visits**

An Interim visit may occur when a study participant, for reasons related to the study, requires evaluation before a scheduled study visit. The Interim visit will be numbered from a study participant's most recent study visit. For example, an Interim visit following study visit 3.0 would be visit 3.1. If a second visit was necessary before the next scheduled Visit 4, it would be visit 3.2 and so forth. A case report form will be completed for each patient at each Interim visit documenting the reason for the interim visit. All patients will be screened for Adverse Events (AEs) at Interim visits.

II.C.10 **Compensation of Study Participants**

Compensation for time and effort put forth by study participants will be offered since the study visit schedule and the assessments and procedures performed for this research will not be integrated with a patient's medical care and will place special demands on a patient beyond those of usual medical care. All study participants will be compensated based on the demands of a clinic study visit and according to the following compensation schedule:

Period	Visit Name/Number	Compensation Amount per Visit
Screening	Screening (Visit 1.0) Period 1	$30.00
Run-in	Run-in Day 0 Period 2 (Visit 2.0)	$40.00
	Week 2 Run-in Period 2 Follow-up (Visit 4.0)	$20.00
	End of Run-in Period 2/Beginning of Period 3 (Visit 5.0)	$40.00
Treatment	Treatment Period 3 Follow-up Weeks 4 and 12 ($20.00 per Visit 8 and 12 in Schedule of Assessment, and 6th and 7th visit respectively in consent document)	$40.00
	Treatment Period 3 Follow-up 24 (Visit 14.0 in schedule of assessments and 9th visit in consent document)	$40.00
	Treatment Period 3 Follow-up Week 36 (Visit 16.0 in schedule of assessments and 11th visit in consent document)	$20.00
	End of Treatment Period 3 Week 48.0 (Visit 18.0 in schedule of assessments and 13th visit in consent document)	$40.00
	Total compensation for all completed clinic visits	$270.00

II.C.11 Outcome Measures

II.C.11.a Co- Primary Outcome Measure

a. Cartilage Thickness by MRI

The study has been designed to determine if PLIUS potentially diminishes articular cartilage thinning by measuring cartilage thickness in the tibial femoral joint. The measure selected to best evaluate this is cartilage thickness of the central medial femoral condyle because studies of the OAI data have shown this region to be among those most sensitive to change [32, 56] and, due to the anatomical positioning of the device, the most likely to be affected by treatment.

The OAI MRI protocol is considered the current standard for MR imaging-based assessment of cartilage OA. We will use a variation of the OAI MRI protocol that eliminates scans of the contralateral knee, eliminates the redundant 3D FLASH acquisition, and adds a secondary DESS acquisition. The secondary DESS acquisition, when averaged with the primary acquisition, will improve SNR and accuracy of cartilage segmentation for measurement of cartilage thickness. Additional physiological data can also be derived from the two DESS acquisitions. Each participating center will be provided with a detailed MRI protocol, (Appendix V.B). A summary of the MRI protocol is summarized in the table below:

Order	Scan	Time
1	3-plane Localizer	1.0
2	Sagittal 3D DESS (water excitation)	10.6
3	Sagittal 3D DESS (water excitation) – modified gradient	10.6
4	Coronal IW TSE FS (3200 29)	3.4
5	Sagittal IW TSE FS (3200 30)	4.7
6	Sagittal T2 Map (120 mm FOV)	10.6
	Total (minutes)	40.9

The protocol as proposed will allow full cartilage morphometry measurements in support of the primary outcome measure of cartilage thickness in the central portion of the central medial femoral condyle, in addition to allowing assessment of maximal size of bone marrow lesions (a secondary outcome measure).

b. OMERACT-OARSI Response

Symptom reduction is the second primary aim of the study. Outcome Measures in Rheumatology Clinical Trials-Osteoarthritis Research Society International (OMERACT-OARSI) Response [11] incorporates changes in pain and global assessment to define response in OA clinical trials. A response is classified as:

1) An improvement in either pain or function of at least 50 percent accompanied by an absolute decrease of at least 20 mm on the visual-analogue scale for pain or function, **OR**
2) The occurrence of at least two of the following: a) a decrease in pain of at least 20 percent accompanied by a decrease of at least 10 mm on the visual-analogue scale; b) an improvement in function of at least 20 percent and a decrease of at least 10 mm on the visual-analogue scale; c) an increase in the patient's global assessment score by at least 20 percent accompanied by a decrease of at least 10 mm on the visual-analogue scale

II.C.11.b **Secondary Outcome Measures**

Secondary outcome measures were selected in accordance with the preliminary recommendations of the Osteoarthritis Research Society task force[82] and include the following:

a. WOMAC Pain, Function Subscales and Total WOMAC Score

The WOMAC® Index questionnaire [33] is a tri-dimensional, disease-specific, self-administered, health status measure. It probes clinically important, patient-relevant symptoms in the areas of pain, stiffness and physical function in patients with osteoarthritis of the hip and/or knee. The WOMAC has been linguistically validated and is a reliable and responsive measure of outcome, and has been used in diverse clinical and interventional environments. The index can be completed in less than five minutes and consists of 24 questions divided into 3 subscales: 1) Pain (5 items): during walking, using stairs, in bed, sitting or lying, and standing; 2) Stiffness (2 items): after first waking and later in the day; and 3) Physical Function (17 items): stair use, rising from sitting, standing, bending, walking, getting in / out of a car, shopping, putting on / taking off socks, rising from bed, lying in bed, getting in / out of bath, sitting, getting on / off toilet, heavy household duties, light household duties.

The Total WOMAC Score, along with the Pain and Function subscales, are secondary outcome measures. The subscale results will be obtained to allow for the possibility that PLIUS may differentially impact measures of OA pain and function. Participants will complete this questionnaire at each clinic visit.

b. Patient Global Assessment of Disease Status

A patient global assessment of disease status on a Visual Analog Scale (VAS) will be measured at each clinic visit. Patients will be asked to quantify their disease status on a 100 mm VAS as follows: "Considering all the ways your arthritis of the

knee affects you, mark ‘X’ on the scale for how well you are doing.” The scale shows the left hand marker “Very Well”, and the right hand marker “Very Poor.”

c. Patient Global Assessment of Response to Therapy

A patient global assessment of response to therapy will be assessed at each post randomization visit. The patient will be asked to rate the overall response of the arthritis in the index knee to the study intervention on a 100 mm VAS as follows: Left hand marker “Excellent-Ideal response, virtually pain free”, right hand marker “None-No good at all-ineffective.”

d. Investigator Global Assessment of Disease Status

An investigator global assessment of disease status on a 100 mm VAS will be measured starting at Visit 2 and at each clinic visit thereafter as follows: “Make a global assessment of the patient's disease status, with regard to the index knee, by marking an ‘X' on the scale below.”, with left hand marker “Very Well” and right hand marker “Very Poor.”

e. Investigator Global Assessment of Response to Therapy

An investigator global assessment of response to therapy will be assessed at each post randomization clinic visit. The investigator will evaluate the patient's response to therapy of the index knee on a 100 mm VAS as follows: Left hand marker “Excellent-Best possible anticipated response, considering the severity and stage of disease”, right hand marker “None-No response, absence of intervention effect.”

f. Knee Evaluation

The site investigator will perform a standardized physical examination of both knees at visit 1 and on the index knee only at visit 2 and thereafter on all patients as modified from the OAI Protocol. The examination will include the following components: visual assessment of knee alignment, anserine bursa tenderness, patellar quadriceps tendonitis/tenderness, crepitus, knee flexion pain, presence of flexion contracture, knee effusions, tibiofemoral joint line tenderness, and patellar tenderness. The objectives of the knee exam are to characterize possible sources of knee pain, assess the severity of selected OA-related knee impairments, identify findings that may correlate with abnormalities detected by MRI (such as knee effusions with synovial enlargement), and evaluate the prognostic value of standard exam findings (i.e., effusion, malalignment and crepitus). Knee exam components were selected based on data demonstrating the potential for reproducibility across examiners [83]. Site investigators will be trained to perform the knee examination (Appendix V.D).

g. Measurement of Intermittent and Constant Osteoarthritis Pain (ICOAP)

This 11-item tool is designed to assess pain in individuals with knee osteoarthritis taking into account both constant and intermittent pain experiences. This tool is designed to be interviewer-administered and takes less than 10 minutes to complete. Study participants will be asked to respond to the questionnaire items based on index knee in the past week (i.e., past 7-day period). Study participants will respond to all questions for the same joint. This tool is intended to be responsive to change in OA pain over time or with treatment.

h. Use of Rescue Analgesic

At each clinic visit patients will return the diary recording all rescue medication (e.g., acetaminophen, tramadol) usage, including name, date, and amount taken daily.

i. Discontinuation of Study Device Due to Adverse Device Event

Safety will be measured by assessing the number of patients who require discontinuation of the study device because of Adverse Device Events (ADE).

j. Bilateral Fixed-Flexion Knee Radiograph for Joint Space Narrowing

At the Day 0 Beginning of Period 3 and the 48-week follow-up visits patients will have a bilateral Fixed-Flexion knee radiograph [8] to assess the status of the medial compartment of the knee (Appendix V.C).

k. Assessment of BML Maximal Size from MRI

Bone marrow lesions (BMLs), as identified on MRI, are associated with articular pain in OA [52]. BML maximal size will be determined from two-dimensional fat saturated fast spin echo MRI sequences acquired in the sagittal plane, as previously described in [84]. Measurements will be made at each MRI scan performed at the Day 0 Beginning of Period 3, and the Week 24 and 48 visits.

l. Biomarkers

OA Biomarker determination provides a potential independent outcome measure. The use of biomarkers to follow patients with OA is rapidly developing [85] and will soon become a standard assessment tool. The combination of MRI and biomarker results has been shown to be effective in identification of OA progressors [86], and urinary Coll 2-1 levels over 1 year were highly predictive of radiographic progression [87]. The ratio of biomarkers associated with cartilage degradation and synthesis has been proposed to differentiate OA stages [88], as has area-under-the-curve (AUC) analysis of serum and urinary markers in combination with imaging, demographic and baseline data. Therefore, collection of soluble biomarkers, especially in combination with our MRI outcomes, may provide important complementary information in the proposed trial. Urine and blood samples will be collected for analysis of: 1) serum

cartilage oligomeric matrix (sCOMP); 2) urine C-terminal telopeptide of type II collagen (uCTXII); 3) a peptide of the alpha-helical region of type II collagen 108HRGYPGLDG116 (Coll 2-1); and 4) a peptide of 9 amino acids nitrated on its tyrosine residue [HRGY(NO2)PGLDG] specific for the type II collagen a1 chain (Coll2-1NO2).

The levels and AUC responses of these metabolites will be analyzed to indicate correspondence with diagnostic severity, prognosis and clinical response. Perhaps most importantly, biomarker response may precede symptomatic, radiographic, and even MRI response to PLIUS. The proposed trial provides a unique opportunity to test the leading soluble biomarkers individually and in combination with imaging outcomes in a prospective fashion as measures of disease modification or progression, adding importantly to the development of biomarkers for OA.

II.D Biostatistical Considerations

II.D.1 Primary Outcome Measures

Since structural modification and symptomatic improvement are considered equally important in OA, two co-primary outcome measures are proposed for this Phase IIa trial: central medial femoral condyle cartilage thickness (cMFCtTh) change from baseline (Day 0, Beginning of Period 3 part of visit 5) to 48-weeks and OMERACT-OARSI response rate (OORR) at 48-weeks. Justification to proceed to a Phase III trial would include demonstrating potential benefit for either outcome measure or both.

II.D.2 Hypothesized Treatment Effects

48-Week cMFCtTh Change Treatment Effect

We will consider PLIUS successful for the cartilage thickness measure if we observe a difference in 48-week cMFCtTh change between sham and PLIUS groups of 33 µm, as realized by a 48-week cMFCtTh change of -14 µm (SD 152 µm) in the PLIUS group (47 µm -14 µm =33 µm) and a 48-week cMFCtTh change of -47 µm (SD 152 µm) in the Sham group. While the treatment attenuated the progression of cartilage degeneration in Guinea pigs in a laboratory setting[5], the effect may be reduced for humans in a clinical trial setting due to less than complete compliance with using the study device or other factors that are less likely to be controlled in a human cohort. We do not expect to reverse progression, but would consider a slowing of progression to be a clinically meaningful result. We assume that the sham group will progress naturally. The assumptions of -47 µm (SD 152 µm) change in the sham group is based on observational data of the natural history of knee OA from the OAI [56] of 1-year cartilage thickness change in the medial femoral condyle region as measured in the sagittal plane using the DESS MRI pulse sequence, with a sample size (n=104) of predominantly KLG 2/3.

48-Week OMERACT-OARSI Response Treatment Effect

We will consider PLIUS successful for the OMERACT-OARSI measure if there is a 10% greater response rate in the 48-week OMERACT-OARSI in the PLIUS group compared to the sham group. In a meta-analysis of 11 placebo-controlled trials of osteoarthritis the average difference in percentage points between the placebo and treatment groups in terms of OMERACT-OARSI responders was 12.7 (95% CI 7.8 – 17.6)[89]. In GAIT, the OMERACT-OARSI response in the placebo and celecoxib groups was 56.9% and 67.3%, respectively, a difference of 10.4% [75].

II.D.3 Ranking and Selection Procedures

Simon et al. [90] first proposed ranking and selection procedures for Phase II trials. The procedure and sample size calculations for continuous normal and binomial outcomes are detailed in Chapters 2 and 4 respectively of Gibbons et al. [91]. A concise summary of the important concepts in this textbook as well as all formulae and tables necessary for sample size calculations are presented in a journal article by the same authors and have been provided [92]. The purpose of the procedure as applied to this study is to identify whether PLIUS has a high probability of being more effective than sham. This is accomplished by obtaining sample estimates of the outcome for the PLIUS and sham groups and determining whether the PLIUS group outcome is better by the pre-specified margin of difference discussed above. The sample sizes accompanying ranking and selection procedures are generally smaller than traditional hypothesis testing procedures. They are established to ensure that if PLIUS is better than sham by amount D then there is a high probability P it is more effective. The magnitude of difference in the sample estimates between the two groups will be considered in the decision of whether or not to recommend a Phase III trial. This process will be used for the structural modification outcome and separately for the OMERACT-OARSI outcome.

Since the ranking and selection procedures are fundamentally different from traditional hypothesis testing, concepts of statistical significance and power have no direct analogue [92]. Furthermore, for the purposes of this Phase II trial, demonstrating potential superiority in either or both primary outcomes would be important. Thus no multiplicity adjustments for co-primary outcomes will be done.

II.D.4 Sample Size Calculation

cMFCtTh Change at 48-Weeks

For the 48-week cMFCtTH change outcome measure the minimally clinically significant difference is 33 µm. With a difference in cMFCtTh measurement of 33 µm, σ=152 µm standard deviation, k=2 groups, and P=0.90 probability of correct selection we use Table 1 of Gibbons et al. 1979 [92] to obtain τ=1.8124, which we use in the equation to estimate the per-group sample size: $n=\sigma^2(\tau/\delta^*)^2=72$ or a total of 144. With a total sample size of 144 the probability of correctly selecting the group with the greatest true cMFCtTh change at 48-weeks is slightly above 0.90 if the true group difference in cMFCtTh change at 48-weeks is at least 33 µm. If the assumed standard deviation of 152 µm turns out to be conservative, realized standard deviations of 196 µm and 237 µm will provide us with probabilities of correct selection of 0.85 and 0.80 respectively.

OMERACT-OARSI Response Rate Difference

For the 48-week OMERACT-OARSI response the minimally clinically significant difference is an absolute rate difference of 10%. We assume a 10% difference in 48-week OORR between sham and PLIUS groups. Using linear interpolation as described in Ranking and Selection (Gibbons el al. 1977)[91] and Table 2 of Gibbons et al. 1979[92], with a total sample size of 144 the probability of correctly selecting the group with the greatest OORR is at least 0.885 assuming (δ=10%) that the sham group is drawn from a population with a 50% response rate and the PLIUS group is drawn from a population with response rate of at least 60% or at most 40% (absolute difference of 10%). The 50% response rate assumption is conservative in that it requires the largest sample size among all possible rates p where $0\%<p<100\%$. In other words, with a total sample size of 144 we can assert with probability at least 0.885 that the treatment effect will be detected if the true difference in OORR is at least 10%.

Adjustment for Withdrawal

We assume an overall withdrawal rate of 20% for the proposed study and will increase the final sample size from 144 to 180 to adjust for this. The strontium renelate OA trial , a 3-year drug intervention trial, reported a withdrawal/exclusion rate of 19% [93]. The Doxycycline OA Trial [10] lasted 30 months and reported withdrawal rate of 29%. In addition to the proposed trial being shorter, it is not a drug trial so we expect fewer withdrawals due to adverse effects. Therefore, we expect the overall withdrawal rate to be lower than the above mentioned trials. 144 subjects is a lower bound required to obtain the probabilities of correct selection stated above, but because we will be using multiple imputation to use some of the withdrawn subjects in the final analysis we will in reality have the ability to detect even smaller treatment effects than previously stated.

Under the current assumptions, the sample size for a Phase III trial to detect a 10% difference in OMERACT-OARSI response rate with a 5% type-I error and 85% power is estimated to be 886. A definitive trial for a cartilage thickness outcome would require a sample size of 816 patients with the same assumptions. Therefore, we do not believe that a

definitive pain study could be sufficiently powered without dramatically increasing the sample size. Most importantly, there is at present insufficient evidence regarding PLIUS treatment in humans in a clinical setting to justify the expenditure required to support a large Phase III trial.

II.E Statistical Analysis Plan

II.E.1 Baseline Characteristics

The randomization will be assessed by comparing the distribution of all baseline characteristics including WOMAC Pain, WOMAC Function, cMFCtTh, JSW, BML maximal size, KLG, BMI, age, race, marital status, education, duration of symptoms, military cohort, and site across the two randomized groups with Wilcoxon rank tests or chi-square tests.

II.E.2 Analyses of Co-Primary Outcomes

cMFCtTh & OORR

Both 48-week cMFCtTh change and 48-week OORRs will be estimated independently and compared in the PLIUS and sham groups. PLIUS-treated will be identified as the group showing a treatment effect if the estimate in the PLIUS group is the larger of the two by the previously specified margins.

Handling of Missing Data

Statistical analyses will be conducted under intent-to-treat principles; specifically, all patients randomized will be used in the final analysis. We estimate about 10% of patients to have no follow-up cMFCtTh data, and about 5% of patients to have less than 12 weeks of OORR data. For these scenarios, group estimates will be obtained using multiple imputation techniques as outlined in Molenberghs and Kenward 2007 Chapter 9 [94].

Multiple imputation will be implemented in SAS PROC MI in two stages. The first imputation stage will use the Markov chain Monte Carlo method (multivariate normal assumption) to produce an imputed dataset with a monotone missing data pattern. The second stage will use the propensity score (nonparametric assumption) method to produce a fully imputed dataset from the monotone-patterned missing data of the first stage. We will create n=5 imputed datasets for analysis and combine the results with PROC MIANALYZE. The 48-week cMFCtTh change will be imputed directly, while the OORR will be calculated from imputed values of 48-week WOMAC pain/stiffness and patient global assessment changes. If a patient elects to receive a total joint replacement of the index knee, their 48-week OORR outcome will be analyzed as “non-responder”.

Multiple imputation procedures produce unbiased estimates under the missing at random (MAR) assumption. MAR means that the dropout can depend on observed data but not unobserved data. Therefore if the chosen imputation model is structurally accurate and includes observed data on all covariates that are relevant to both the outcomes and dropout, then estimates calculated from the imputed datasets are unbiased. Since all randomized patients will have baseline and follow-up data, we can reasonably assume that the MAR assumption will be met if we include all available information in the imputation model. The model used to impute missing data will include baseline covariates listed in Section II.E.1 as well as any WOMAC pain/stiffness, patient global assessment and cMFCtTh outcome data available through 48-weeks. Compliance data will also be included in the imputation model as it will likely be predictive of dropout, and therefore important in meeting the MAR assumption [94]. However, to provide additional assurance we will perform a sensitivity analysis for the primary outcomes in which we will vary the relationship between the mean of the imputed missing outcome data and dropout and compare the resulting estimates to assess the MAR assumption.

II.E.3 **Analyses of Secondary Outcome Measures and Secondary Analyses of Primary Outcome Measures**

The continuous outcome measures cMFCtTh, Fixed-Flexion view JSW, BML maximal size, WOMAC Total, WOMAC Pain subscale, WOMAC Function subscale, ICOAP total pain score, Patient/Physician Global Assessment of Disease Status and Response to therapy will be analyzed as follows: 1.) A mixed-effects linear regression model, as outlined in Hedeker and Gibbons Chapter 9 [95], using all available data over the 48-week study period will be fit to determine group differences in rate of change over time.**†** 2.) In addition, mean change from baseline to 24 and 48 weeks and the associated standard deviation will be calculated for each group. 95% confidence intervals will be calculated and a t-test (α=0.05) for statistical significance will be performed. 3.) ANCOVA will also be performed (α=0.05) on continuous measures at 48-weeks, adjusting for baseline measures mentioned in Section II.E.1. 4.) For the WOMAC pain and function subscale mean change from baseline to 48-weeks, as well as a 1-way MANCOVA with and without adjusting for baseline measures as described in Section II.E.1 will be performed, as will a test for overall group differences (α=0.05).

The categorical outcome measures OORR and standardized physical examination measures will be analyzed as follows: 1.) A mixed-effects logistic regression model, as outlined in Hedeker and Gibbons 2007 Chapter 4 [95], using all available data over the 48-week study period will be fit to determine group differences in response rate over time.**†** 2.) A Chi-square (α=0.05) test of the 48-week response rates will be performed. Proportions, standard deviations, and 95% confidence intervals will be reported at each time point. 3.) Logistic regression will also be performed (α=0.05) on the above measures at 48-weeks, adjusting for baseline measurements as described in Section II.E.1. 4.) Longitudinal mixed-

effects logistic regression analysis will be performed using outcomes at 4, 8, 12, 24, 36 and 48 weeks as per Hedeker and Gibbons 2007, chapter 9 [95] for binary OORR.

Device Usage Compliance

Descriptive statistics of the percent compliant over time will be calculated as the total number of treatments administered (as reported by the device) divided by the number of days the device was available in the patient's home since the last clinical visit X 100. This measure, collected every visit over 48 weeks, will be treated as continuous data and as such point estimates and confidence intervals will be reported for each time interval as well as cumulatively up to the end of the study for both groups separately and combined. A mixed-effects linear regression model, as outlined in Hedeker and Gibbons 2007 Chapter 4 [95], using all available data over the randomized 48 week study period will be fit to determine group differences in compliance rate over time.†

† (H_0:β(treatment x time interaction)=0) Fixed-effects will include: intercept, time, treatment, treatment x time. Random-effects will include: intercept and time.

II.F **Stratified Analyses**

To account for potential confounding due to intermittent use of NSAIDs or site-to-site variability we propose two stratified analyses based on (1) intermittent use of NSAIDs and (2) study site. Intermittent NSAID use will be defined as less than 30 days of continuous use at a stable dose. Stratified analyses will be performed for each of the co-primary outcomes. As discussed in Section II.D.3, the concepts of statistical significance and power have no direct analogue; subsequently, attempts to formally test heterogeneity (e.g., examining model-based interactions, Cochran-Mantel-Haenszel) may suffer from low statistical power. Therefore, descriptive statistics (e.g., mean, median, standard deviation for continuous outcomes and proportions for categorical outcomes) and plots (e.g., mean for continuous outcomes and proportions for categorical outcomes) of the co-primary outcomes by treatment group will be reported for each strata. If heterogeneity in primary outcomes is suspected across the strata, further assessment of sources of variability, including, sampling factors and study protocol characteristics, will be conducted [99].

II.G **Site Performance Monitoring**

The VA San Diego Health Care System (VASDHCS), the VA North Texas Health Care System (VANTHCS) and the VA Salt Lake City Health Care System (VASLCHCS) are the three participating centers for this study. The Hines VA CSPCC will evaluate each center's recruitment and retention performance monthly. Other performance problems such as protocol

deviations, poor data quality, missing or overdue data, and reasons for withdrawal from study will also be tracked. A study conference call will be held monthly for study personnel including Hines VA CSPCC, CSPCRPCC, the Study Chair's office, and all sites to review study recruitment, data quality, and protocol adherence until the last enrolled study participant has completed the follow-up visit. Conference calls may be more frequent if study leadership decides it would be advantageous. Study sites will be put on probation for poor performance, including under-recruitment. Typically the probationary period is three months, at which time the site may be taken off probation, have probation continued, or have funding reduced or stopped, depending on its performance during the probationary period. The Hines CSPCC Director is authorized to make those recommendations to the CSR&D Director and seek approval. Should the site have funding stopped, an alternate site will be selected.

II.H **Data Collection and Quality Control Measures**

The Hines CSPCC will be responsible for the management and the quality control of the data. DataFax, a clinical trial data management system (by Clinical DataFax Systems, Inc.), will be used for data collection and management. DataFax allows for paper data form collection as well as electronic data capture (EDC). After a patient consents to participate in the study, the site coordinator will create a patient casebook, which will contain the consent forms, all relevant source documents, and any other information pertinent to the study. The Study Coordinators from the sites will complete case report forms (CRFs) on a daily basis and fax them directly to the DataFax computer server, where data images of the CRFs are stored as files. The original forms will be kept in the investigator's study files. The DataFax system uses an optical character recognition (OCR) paradigm to automatically process and store the information from the image as data into the study database. The original fax image is also stored. Data management staff at CSPCC will review each CRF by comparing the faxed image with the OCR data and ensure that the two match. If in the future the data capture process changes to electronic CRFs, the Study Coordinators will log into the web-based DataFax system and enter study data directly, rather than completing and faxing a paper CRF.

Data management staff at CSPCC will review CRFs for protocol adherence data consistency, and add data queries to items that fail these checks. Checks will be performed manually and programmatically. On a regular basis, data management staff will produce site-specific Quality Control reports that list all unresolved data queries. Data management staff will make the reports available to each site and work with the Study Coordinators to help them resolve queries. Queries will be resolved when the appropriate corrections to the CRF are made and data resent, or when an explanation is provided that allows for data management staff to resolve the query. All corrections and changes to the data will be reviewed by data management staff. In addition to the Quality Control report, CSPCC may generate and distribute targeted data edit reports on an as needed basis.

Study site personnel will be provided with an Operations Manual to guide them through the operation and management of the study and data collection tools. Study personnel will receive training at a kick-off meeting to assure uniformity in patient management, data collection, study procedures, and Good Clinical Practices. All participating investigators, site research coordinators, and CSP coordinating center personnel will be in attendance. The Study Chair's office and the data (Hines VA CSPCC) and pharmacy coordinating (CRPCSPCC) centers will provide the training. The general training will include the study device, patient screening and consent, baseline evaluation, follow-up procedures, proper collection and maintenance of data, and ADE/AE/SAE safety reporting.

At this training, the Study Coordinators will be provided with instruction and reference materials on the case report form completion. Formal training on case report forms for clinical study management will also be provided. Training will also be held at annual meetings and on an as-needed basis for new study personnel.

II.I MRI Acquisition and Quality Control Measures

The use of a quantitative cartilage morphometry measure in MRI such as the cartilage thickness outcome requires close attention to image acquisition consistency, image quality, and analysis consistency. Standardization of the MRI acquisition procedures and analysis across sites is critical to the study's success, and has been carefully considered in the study design. We have assembled a team of OA MR imaging experts to develop and execute the MR protocol. A Salt Lake City based MR image team will perform all MRI analyses (including image segmentation, quantitative measurements, and semi-quantitative measures). The study is based on identifying longitudinal changes across the treatment period in individual study participants who will be imaged on the same MRI hardware at each imaging point, mitigating problems arising from inconsistencies across hardware platforms. The chance of any such difficulties arising is greatly minimized by the fact that each participating site uses a Siemens 3 Tesla MRI system, similar to those extensively validated in the OAI. Analyses will be performed at the end of each patient's participation in the study. The reader will be blinded to time point and the participant's images will be segmented simultaneously to ensure intrasubject consistency. Test-retest reliability will be calculated at each time point by sampling 20 images. The reader will analyze each image twice, 7 days apart, followed by calculation of Pearson's correlation coefficient. Drs. Schneider and Kwoh, both of whom were extensively involved in MRI data collection and quality assurance measures for the OAI, will work closely with Drs. Clegg and Bangerter to implement the image acquisition and MRI protocols, as well as ongoing QA procedures. Images will be sent electronically to the study's Salt Lake City VA based MR imaging team using a VA-approved transmission process. Site personnel will be trained in image acquisition, MR coil usage, patient positioning, QA and procedures at each site. The SLC-based imaging team will also monitor QA phantom and patient images monthly from each site to ensure suitability for analysis.

The complete MR imaging protocol will also be validated at each of the recruitment/imaging sites, both on phantoms to assess image quality across sites and in vivo, prior to commencement of the study. Scan operators at each site will be trained to exactly follow the prescribed protocol, ensure consistency of study participant positioning in the scanner, and visually inspect images as they become available during the scan for image quality and identification of potentially problematic aliasing or other image artifacts. Scan operators at each site will provide the sample MR images monthly for evaluation and control by the SLC-based imaging team. Quality assurance measures can be found in the study's MR Imaging Protocol (Appendix V.A)

As mentioned, all participating centers have Siemens 3 Tesla MRI systems. This platform has been extensively validated by the OAI for the MRI-based outcome measures proposed in this study. The DESS contrast needed for morphologic measurements of cartilage is easily and consistently achieved on this platform, and the proposed study will further analyze longitudinal changes within individuals, each of whom will be scanned at all time points on a single scanner. We have considered this point very carefully in the study design, and feel the team is well qualified and equipped to ensure that each image acquisition site will yield consistent data of sufficient quality for the proposed analysis.

II.J **Bilateral PA View Fixed-Flexion Knee Radiograph Acquisition and Quality Control Measures**

Bilateral Fixed-Flexion knee radiographs will be obtained at the Screening, Day 0 of Period 3 and Week 48 visits. A radiographic imaging protocol will be provided to each center (Appendix V.C). In summary, the radiographs will be obtained in PA projection and 20-30 degrees of flexion with feet externally rotated 10 degrees; right and left knees will be imaged together on 14/17 inch film using a focus-to-film-distance of 72 inches, and knee flexion and root rotation will be determined for each study participant using a plexiglass positioning frame (Synaflexer™) provided to each center. Each participating center will need to dedicate at a minimum a primary and a back-up radiology technician who have been certified through training to perform the radiographic imaging protocol. Any new personnel will be required to complete training before performing radiographs.

Digital radiographic equipment will be used and will need to meet the requirements of the imaging protocol and pass an initial evaluation of image quality. All radiographic equipment used in this study must be maintained on an ongoing basis and according to a regular quality assurance program. Each study center will attempt to identify a single x-ray unit that can be used for all study radiographs for the duration of the study to avoid an unnecessary source of variability.

Knee radiographs will be sent electronically using a VA-approved transmission process to the study's Radiology Coordinating Center located at the VASLC Radiology Department and will be read by one musculoskeletal radiologist

blinded to identifying information about the study participants. The central radiologist will also grade X-rays and make the final determination on X-ray eligibility based on the Screening Fixed-Flexion X-ray[8]. The central radiologist will record the presence or absence of each of the following radiographic features: tibiofemoral joint space narrowing, osteophytes, subchondral bony sclerosis, and valgus deformity of greater than 10 degrees. Joint space width will be determined by the central reader blinded to study participant identity and order of images.

To avoid drift in radiology scoring, the primary reviewer will annually rescore a random sample (approximately 20) of radiographs obtained during the previous year. Agreement with the previous reading of these teaching radiographs must be 85% before new radiographs are scored. Joint space width will be determined by the central reader blinded to study participant identity and order of images.

It is expected that the majority of the radiographs will be of acceptable quality. Ideally, radiographs of insufficient quality should be identified by the radiology technologist at the time of acquisition and repeated immediately. If any problems are detected, however, the Radiology Coordinating Center will notify both the responsible site and the Hines CSPCC, suggest possible causes of the problem, and offer potential solutions to assist the site in producing a good quality repeat exam.

II.K General Guideline for Administration of Study Participant Questionnaires

All participant questionnaires are self-administered and will be done at the start of each clinic visit before any other procedures are performed. Study participants will be instructed to complete all the self-administered questionnaires without any assistance from study staff. Study staff should not assist study participants in answering any questions. Study staff will check that the patient has completed all questions before they leave the clinic.

II.L Administration of the WOMAC Questionnaire

The WOMAC index is self-administered and does not require the presence of a study coordinator. Study staff will read instructions for completing the questionnaire to each study participant before they answer any questions at Screening Visit 1.0. Study participants will be instructed to answer WOMAC questions considering their symptoms over the preceding 24 hours. Patients may hear instructions as frequently as needed.

II.M Exogen Device Quality Assurance

The CRPCC and study sites will maintain the Exogen PLIUS devices supplied provided by Bioventus under the manufacturer's recommended temperature and humidity storage conditions (32° to 122° Fahrenheit and 30% to 75% relative humidity). These conditions are achievable at the selected study sites and participants' homes. The device does

not have any user serviceable or internally accessible parts, which minimizes the risk for output configuration changes. Importantly, the FDA classified the device during its development as a class III device, which required a Pre-Market Approval application for marketing clearance. Manufacturers of approved class III devices must establish and follow quality systems to help ensure that their products consistently meet applicable requirements and specifications. Consequently, class III devices are subject to the strictest level of post-marketing regulatory control and quality. Accordingly, Bioventus maintains the device under a full-scope Quality Management System. This system meets the requirements of U.S. 21 CFR part 820 "Quality System Regulation," as well as certification to ISO 13485:2003 "Comprehensive Quality System for the Design and Manufacture of Medical Devices."

II.N **Study Organizational Structure**

The groups charged with centrally monitoring the various aspects of the study will be: the Executive Committee (EC) and the independent Data Monitoring Committee (DMC). For information on the DMC, refer to section III.G. Both committees meet at study start-up and annually thereafter. The EC will also have quarterly conference calls. The EC and DMC may elect to meet more frequently if deemed necessary.

The EC is the management and decision-making group for the operational aspects of the study. One of its major responsibilities is to monitor the performance of the participating medical centers. The EC considers the need for protocol modifications. The EC also reviews and approves all manuscripts and abstracts emerging from the study. The EC will comprise the original study Planning Committee and will be chaired by the Study Chairman.

The Study Group, which consists of all participating investigators and study personnel at the Hines CSPCC and the Albuquerque CSPCRPCC, will meet annually to discuss the progress of the study and any problems encountered during the conduct of the trial. Finally, the study protocol and progress must be reviewed annually by the local IRB and the local R&D Committees at each participating study site.

III. Human Subjects

III.A **Risk to Participants**

III.A.1 **Human Participant Involvement and Characteristics**

Eligible male or female participants will be at least 40 years of age and have clinical and radiographic evidence of osteoarthritis of the knee. Participants will need to have a summed pain score of 125mm to 400mm on the index (more symptomatic) knee according to the Western Ontario and McMaster Universities Osteoarthritis Index and be in American Rheumatism Association Functional Class I, II, or III. Patients will be excluded if they have concurrent medical or arthritic conditions that could confound evaluation of the index joint, predominant patellofemoral disease, a history of clinically significant trauma or injury to the index knee requiring surgical repair, or coexisting disease that could

preclude successful completion of the trial. All participants will be evaluated at the Screening visit to determine whether they meet entry criteria and are eligible to participate in the Prerandomziation 4 Week Sham Run-in Period. Complete inclusion and exclusion criteria are contained in Section II.C.2.b. Subjects not meeting entry criteria will be excluded. The Run-in Period will consist of a follow-up telephone call for treatment compliance, a follow-up clinic visit (may be conducted by telephone, refer to section II.C.6 for details), and an End of Run-in clinic visit at weeks 1, 2 and 4, respectively, after the Run-in Day 0 visit. Participants will be withdrawn from the Run-in Period if they are less than 60% [28] compliant with the daily treatment regimen at week 2 and less than 80% compliant at week 4. Participants who successfully complete the Run-in Period will be evaluated at the Day 0 Period 3 part of visit 5 to confirm they are eligible to be randomized into the Sham-controlled Treatment Period 3 of the study. Eligible participants will be randomly assigned to the PLIUS or sham device. During the Run-in and Treatment Periods participants will apply the study device for one treatment period of twenty-minutes each day. Participants will be allowed to take a stable dose of NSAID, and acetaminophen or tramadol as rescue analgesia for the relief of uncontrolled knee pain. Participants will be asked to avoid NSAID and rescue medication use during the 24 hours before a clinical evaluation for joint pain.

During the 48-week Sham-controlled Treatment Period 3, participants will be evaluated at Day 0, and at 4, 12, 24, 36 and 48 weeks, and will receive follow-up phone calls for treatment compliance, other analgesic use, and AE/ADE review at 18, 30 and 42 weeks after randomization.

Pregnant women will not be eligible to participate since the safety and effectiveness of the use of this device for pregnant women is unknown. Economically and educationally disadvantaged, homeless, employees, and students will not be directly recruited but will not be excluded if they meet entry criteria and wish to participate. Other vulnerable populations, such as children, prisoners, institutionalized individuals or individuals with decisional impairment will not be allowed to participate.

Compensation for time and effort expended by study participants will be offered since the study visit schedule and the assessments and procedures performed for this research will not be integrated with a participant's medical care and will place special demands on a participant beyond those of usual medical care. A participant who completes the entire study could be compensated up to $270.00. A detailed per visit compensation schedule is in section II.C.10.

III.A.2 Potential Risk

Participants will be at minimal risk. The PLIUS device that will be used in this study is a commercially available FDA-approved device indicated for the non-invasive treatment of non-union bone fractures and for accelerating healing time

of fresh fractures. The PLIUS system consists of one main operating unit, gel bottles and strap. The main operating unit provides the treatment control circuitry, the primary battery supply, and monitors the operation of the transducer at the treatment site. The signal specifications cannot be altered. The sham and PLIUS devices will appear and operate similarly, except no ultrasound signal will be generated by the main operating unit of the sham devices. The PLIUS device is incapable of producing harmful temperature increases in body tissue. The output intensity of the device the participant receives is 30 mW/cm^2 and is typically only 1% to 5% of the output intensity of conventional therapeutic ultrasound devices. The ultrasound intensity is comparable to diagnostic ultrasound (1 to 50 mW/cm^2), such as the intensities used in obstetrical sonogram procedures (fetal monitoring). In addition, there is no evidence of non-thermal adverse effects (cavitation).

The device (called the Sonic Accelerated Fracture Healing System or SAFHS) selected for use in this study to deliver the low intensity ultrasound dose (30 mW/cm^2 delivered daily over 20 minutes) is FDA-approved for the treatment of non-union bone fractures. Therefore, the Albuquerque Clinical Research Pharmacy Coordinating Center (CRPCC) filed an Investigational Device Exemption (IDE) application with the FDA on the basis that promotion of cartilage growth via PLIUS is currently not an approved indication for the Exogen device in the U.S. The FDA granted approval of the IDE application number G130292 on May 28, 2014. Bioventus, the company that manufacturers the SAFHS, follows U.S. 21 CFR part 820 Subpart C “Design Controls & Quality System Regulation” and ISO certification 13485:2003 “Comprehensive Quality System” for the design and manufacture of medical devices. This system ensures the Exogen device remains safe and effective during its intended use and includes broad-scope activities such as design, specification and supplier controls, training, complaints and risk management. The FDA classified the Exogen device as “Class III” device, which means the device must undergo a pre-market FDA approval and is subject to the strictest level of regulatory controls both during development and following market release.

Participants will administer treatment once daily for 20 minutes. The operation of active, implantable devices, such as cardiac pacemakers, may be adversely altered by close exposure to the Exogen device. Participants with a cardiac pacemaker will not be eligible to participate in the study. There are no other known contraindications to the use of the PLIUS device and no device related adverse reactions or medical complications related to the use of the PLIUS device have been reported during non-union bone fracture clinical trials. Some participants have experienced mild skin irritation caused by skin sensitivity to the coupling gel. Resolution can be obtained by a change of coupling medium to mineral oil or glycerin. While participants are expected to be at minimal risk in this trial and the safety of the use of this device has been demonstrated in non-union bone fracture clinical trials in subjects followed over a period of 6.5 years, participants in this trial will be carefully monitored for Adverse Device Events (ADEs).

There is a small risk that by participating in the study the participant's confidentiality may be breached or the participant's protected health information, whether electronic or paper based, may be stolen. There may be risks that are currently unforeseeable.

III.A.3 **Sources of Materials**

Research material will be obtained from participants' existing medical records and data collected from assessments, questionnaires, knee MRIs and X-rays, urine and blood specimens, and all other procedures performed as described in Section II.C of this application. Information from existing medical records and data collected during this trial will be obtained specifically for research purposes.

We plan to collect urine and blood samples from all participants for OA biomarkers analyses. OA biomarkers have potential to assist in the clinical management of patients with OA for diagnostic, prognostic, and response-to-treatment assessment purposes. Planned biomarker analyses in this research are: cartilage oligomeric matrix protein (COMP), collagen fragments in urine (uCTX-II), a peptide of the alpha-helical region of type II collagen 108HRGYPGLDG116 (Coll 2-1), and a peptide of 9 amino acids nitrated on its tyrosine residue [HRGY(NO2)PGLDG] specific for the type II collagen a1 chain (Coll2-1NO2).

III.A.4 **Therapeutic Risks**

No therapeutic risks are associated with this investigational device intervention as all procedures for this trial will be performed for the purpose of this research project and would not normally be performed as part of a person's routine medical care.

III.A.5 **Research Risk**

Several interventions will be performed for the purposes of this research project and all interventions are necessary to achieve the aims of the research. There are no alternative procedures available that will provide the needed data. Participants would not normally have these interventions as part of their routine medical care. Participants who complete all visits in this trial will have these research interventions: MRI of index knee at the Day 0 Period 3, Week 24 and Week 48 visits; Fixed-Flexion x-ray of the knees at the Screening visit, Day 0 Period 3 and Week 48 visits.

The risk associated with an MRI of the knee is very minimal. MRI has no known long-term risks. The MRI machine is a large magnet and could move metal-containing objects in the body. As part of the screening process and prior to

enrollment in the trial, participants will be screened for contraindications to MRI scanning and will be asked questions about their medical history. If a participant has a piece of metal in their body, such as a fragment in the eye, aneurysm clips, ear implants, spinal nerve stimulators, or a pacemaker, they will not be able to receive an MRI. Participants with known contraindications to MRI scanning will not be eligible to participating in the trial. Ear protection will be provided to participants since the MRI produces repetitive tapping noises resulting from normal operation of the scanner.

Some participants may be claustrophobic and experience fear when confined to a small space. Participants will be asked if they have had an MRI and were given pre-medication to help with anxiety or claustrophobia. Arrangements for pre-medication will be made and administered by the radiologist prior to the MRI.

This research study involves X-rays of the knees and exposure to ionizing radiation. The risk of these procedures is small. The amount of radiation exposure participants will receive from these procedures is very low.

Participants will have their blood drawn for collection of biomarker samples at Day 0 Period 3, and at 12, 24 and 48 weeks after randomization. Needle punctures carry some risk such as fainting, bleeding, bruising, discomfort, dizziness, infection and pain at the point of puncture site.

III.B **Adequacy of Protection Risk**

III.B.1 **Recruitment and informed consent**

Participant recruitment will occur at each participating center. The recruitment period will last approximately 23-months. Potential participants will be identified and recruited using a number of strategies as described in Section II.C.2. Participants identified through the aforementioned recruitment methods will have their medical history pre-screened by study staff to determine potential eligibility per inclusion and exclusion criteria. Potentially eligible participants will be informed of the study by their primary or specialty care providers and instructed on how to contact study staff if interested in participating. Interested individuals will be contacted by phone to discuss the research project, what participation will involve, and to review their eligibility to participate in the study. If potentially eligible based on the phone screen, the patient will be scheduled for a Screening visit at the clinic and will be mailed a consent document. Patients will be asked to read the consent document prior to appearing for their Screening visit, to discuss the study with family or friends if they wish, and to take time to decide whether they want to participate. They will also be encouraged to contact study staff if they have any questions.

The investigators or their delegated research staff will be responsible for obtaining consent. The purpose of the consent process will be explained to the patient at the onset of the Screening visit and the consent document will serve as the basis for a meaningful exchange between the research staff and the patient. Consent will be obtained before any study-related procedure is performed. The patient will have a copy of the consent document to follow and reference during the consent process. All elements of the consent document will be reviewed with the patient and they will be encouraged to ask questions. They will also be asked questions to verbalize understanding of the risks, why the study is being done and what it will involve. To minimize the possibility of coercion, patients will be encouraged to take as much time as they like to consider participating and will be told no matter what they decide to do their decision will not affect their medical care.

A signed copy of the consent document will be provided to the participant and the original signed consent document will be retained in the study records. The original consent form will be kept in locked file cabinets within locked offices and referenced by participant ID number.

III.B.2 **Protection Against Risk**

The risks of the investigational intervention, procedures and participation in this trial are minimal. Nonetheless, each participant’s health and well-being will be the primary concern of the research team. Participants will be monitored for any adverse experiences and unanticipated problems involving risks to participants, whether or not considered related to a study procedure. All participants presenting to the VA medical center will be provided treatment when appropriate for all AEs, UADEs and SAEs that occur during the study period. Participant privacy and confidentiality and data security risks are minimal in this research project. Protections and precautions will be taken to ensure risks are minimized. The following precautions will be used to ensure privacy is maintained: research procedures will be conducted in private clinic rooms or appropriate ancillary care facilities; only those authorized to conduct study-related procedures will be permitted in patient care areas unless express permission is given from the participant for other persons to be present; the study will be discussed with participants individually instead of in front of a group; and the collection of information about participants will be limited to the amount necessary to achieve the aims of the research so that no unneeded information is being collected.

Handling and storage of study data will adhere to current VA policies. The database will not contain information that can directly identify the study participant (such as name, address, etc.); however, it will not be a completely de-identified database since age and study visit dates will be collected. The Hines CSPCC requires that a copy of the signed consent form, HIPAA waiver, and a participant contact sheet be on file at CSPCC. The consent form is required by CSP

policy in order for the coordinating center to independently certify that all study participants have provided written informed consent. The consent process is needed in order for participants to be screened and participate in the study. Because Hines VA CSPCC is the final data repository for the study, participant contact sheets are collected in the event study participants need to be contacted (such as safety notices) after study sites have completed the study. The CSPCC is the final repository of all study data and the only component that remains open and funded after the study ends. In the event that a study participant should need to be contacted well after the end of the study, his or her name, address, and phone number will be vital to the CSP center. Each participant's SSN is also necessary in the event that the Coordinating Center should have to access the Beneficiary Identification Records Locator (BIRLS) files to locate a participant. The electronic information from the contact sheets is stored separately from the main study database in a password-protected file. Consent forms and contact form information will be uploaded or faxed into the electronic system as password-protected files.

Two mirror-images of the study database will be housed on separate servers located at different secure VA facilities that support round-the-clock web services and monitoring within a secure VA environment in order to provide an optimal infrastructure for the protection of sensitive information. Data entered by the site coordinator is sent to the production server which is then copied almost continuously to a back-up server at a different location. The clinical database with all research data will be housed behind the VA firewall on VA-owned and maintained servers. Accordingly, the information housed within the DataFax system will be afforded the same level of security as all forms of VA protected and/or highly sensitive information. Additionally, the system will be monitored by the Hines VA CSPCC Quality Assurance and Information Technology teams to ensure that all applicable VA regulations and directives are strictly followed.

Backup copies of the database will be transferred behind the VA firewall to the Hines CSPCC on a frequent basis depending on the study need (at least once per day). These backup copies will be transferred and stored across secure connections according to VA regulations and Hines CSPCC operating procedures. Periodic off-site back-ups will be made as part of a comprehensive disaster recovery plan. The Data Management group will ensure that backup media are stored in compliance with all federal and VA regulations on the storage of potentially sensitive information. The Data Management group will also ensure that all backup media is encrypted in compliance with the current best practices established and approved by the Center Director(s). Encrypted backup media will be stored in a physically secure location with access restricted to essential personnel. Access to back-ups may be at the discretion of the Data Management group.

Access to the study data is heavily restricted to individuals with CSP approval. Individuals must be properly credentialed research staffs who are compliant with VA security trainings (i.e., Research Data Security, HIPAA and VA Privacy Training,

Information Security Awareness, and Good Clinical Practices). In addition, research data will be stored on VA secure servers with restricted permissions for copying and exporting data. Only properly approved coordinating center personnel will have the ability to copy and export data. These individuals have received training on the local standard operating procedures (SOP) governing their permissions and will not access or export data without approval from the Hines CSPCC Center Director. Furthermore, the permissions of the electronic systems are structured such that individual sites can only see the data for their study participants. They cannot see or access the data for another clinical site or for another participant.

Access to protected health information (PHI) will be heavily restricted to individuals approved by CSP to have access to the data.

At the local clinical sites, the staff positions that have access to PHI include: Site Principal Investigator, Site Co-Investigator, and Study Coordinators. Individuals in these positions will be able to access all forms of PHI.

At the Hines CSPCC, the staff positions that have access to PHI include: Project Manager, Biostatistician, Data Management Programmer, Quality Assurance Officer, Quality Assurance Nurse Specialist, Data Coordinator and Statistical Programmer. Individuals in these positions will be able to access all forms of PHI.

At the CSPCRPCC, the staff positions that have access to PHI include: clinical monitors and Adverse Event Specialist (Regulatory Affairs and Safety Officer). Individuals in these positions will be able to access de-identified forms of PHI.
So that research staff understand and are able to implement risk minimizing measures, VA and VA-WOC employees will be required to completed regular training such as Data and Information Security, Privacy, HIPAA, Human Subjects Research Protection, and Good Clinical Practices.

III.C **Potential Benefits of Research to Participants**

The risks to participants are minimal and are reasonable compared to the potential benefits of the knowledge that will be gained from this research project. We hope PLIUS demonstrates potential as a therapeutic modality for knee OA in humans. While there may be no direct benefit to participants taking part in this study, we hope that our improved understanding of the biology of OA gained through this research will benefit others in the future.

III.D Importance of Knowledge to be Gained

The importance of knowledge to be gained is significant and the minimal risks to participants are reasonable in relation to the importance of the knowledge that reasonably may be expected to result. Knee OA is a leading cause of disability [1] in the US and is seen in even higher frequency among individuals with military service [2]. Current therapies are modestly effective at best and are primarily directed at pain relief. There are no FDA-approved interventions that have been shown to impact the progression of OA[3].

In this protocol we have presented consistent and striking evidence for the salutary effects of PLIUS on chondrocyte metabolism and on cartilage, including its effect to forestall the onset of OA and to limit its progression after establishment of disease in an animal model of human idiopathic OA. This research project is an exploratory evaluation of PLIUS as a potential therapeutic modality for knee OA in humans. If PLIUS demonstrates potential as a disease and symptom modifying intervention for idiopathic OA of the knee, it would remarkably advance the understanding of the biology of OA and serve as strong endorsement for a pivotal trial designed to rigorously investigate PLIUS as a treatment that could delay the progression of a debilitating and ultimately incapacitating disease.

III.E Data Monitoring and Reporting

III.E.1 Data Monitoring

The Quality Assurance section at Hines CSPCC will assist in centralized monitoring and remote-based methods of monitoring of study sites to ensure compliance with the protocol, Good Clinical Practice (ICH E6) and VA regulations. Monitoring may include, but not be limited to; the informed consent process, regulatory documentation review, source document verification, and safety reporting. Additionally, study and site metrics will be used as triggered monitoring actions, as appropriate. Site performance concerns may result in on-site visits by the CSP S.M.A.R.T. group and/or the QA Nurse Specialist to evaluate the sites' need for additional training to remedy any compliance concerns. SMART (Site Monitoring, Auditing, and Resource Team), a division of the CSP Clinical Research Pharmacy Coordinating Center (CSPCRPCC), may conduct full audits of participating sites if requested by the sponsor (VA CSRD).

VA study personnel will adhere to all ORD policies on human subjects' protections by fully completing approved required Good Clinical Practices (GCP) training per VHA Handbook 1200.05. Additionally, completed training certificates will be provided by study personnel to the Hines Coordinating Center to maintain on file throughout the study.

The Data Monitoring Committee (DMC) will also review the progress of the study, including patient recruitment, participant compliance,completeness of follow-up, data quality, protocol deviations, and safety. For more information on the DMC, refer to section III.G.

III.E.2 Data Reporting

The Study Co-Chairs, the Site Investigators and Site Coordinators will receive biannual reports regarding the quality and quantity of data submitted to the CSPCC. Other quality control measures include periodic reports containing participant recruitment information and relevant medical data for review by the Study Co-Chairs. The CSPCC will also prepare summary reports for the Study Co-Chairs, the Data Monitoring Committee, and other monitoring groups to track progress, and conduct final analyses of the study data.

Study reports will be generated using DataFax, SAS, and other tools (e.g., Microsoft Excel and Access). SAS and other statistical software packages will be used to conduct data analysis for the study. The CSPCC is using SAS Version 9.3 and will upgrade to newer versions once they are purchased and validated.

III.F Safety Monitoring and Reporting

III.F.1 Safety Monitoring

Study participants will be monitored at each visit for Adverse Events (AEs). Adverse Events include Adverse Device Events (ADEs), non-device related Adverse Events and Serious Adverse Events (SAEs). Adverse Device Events are further categorized into Adverse Reactions (ARs), Suspected Adverse Reactions (SARs), and Unanticipated Adverse Device Events (UADEs). Monitoring and collection of AEs will begin at the time informed consent is obtained. All AEs (including SAEs) regardless of their relationship to the study and ADEs will be collected and recorded on the appropriate study event forms. New AEs and ADEs will be assessed at follow-up and interim visits. In addition, all unresolved ADEs and SAEs will be followed at a minimum every 30 days until 1) resolution of the event or 2) the event resolves with sequalea and the participant is clinically stable. The every-30 day follow up will be conducted by phone or routine clinic visit, whichever permits adherence to an every-30-day follow up schedule. Participants with unresolved ADEs or SAEs at study completion or early termination will be contacted by phone 30 days after their termination from, or completion of, the study. Investigators will ensure that participants with unresolved ADEs or SAEs requiring follow-up care 30 days past study completion or early termination are referred for appropriate follow-up care. Each investigator will record and report all SAEs they are made aware of that occur within 30 days of a participant’s completion of or early termination from the study.

The study intervention is the PLIUS ultrasound device as described in this proposal. The operation of active, implantable devices, such as cardiac pacemakers, may be adversely altered by close exposure to the Exogen device. Participants with a cardiac pacemaker will not be eligible to participate in the study. There are no other known contraindications to the use of the PLIUS device and no device related adverse reactions or medical complications related to the use of the PLIUS device have been reported during non-union bone fracture clinical trials. Based on this we do not anticipate an investigator would withdraw a study participant due to an Adverse Device Event.

III.F.1.a **Role of the Local Site Investigator in Safety Monitoring**

The local site investigator will be responsible for following these safety reporting requirements:

a. Reviewing the accuracy and completeness of all reported AEs, UADEs and SAEs prior to submission in the study database
b. Complying with study/IRB policies as well as the Operations Manual for the reporting of AEs, UADEs, SAEs, Events of Special Concerns and protocol deviations
c. Reporting to the IRB safety issues reported to the site by the sponsor.
d. Reporting UADEs, unanticipated SAEs and unanticipated problems to the local IRB
e. Grading AEs in terms of their severity and attributability using Operations Manual definitions
f. Closely monitoring study participants for any new AEs
g. Following unresolved ADEs and SAEs every 30 days until resolution or resolution with sequaleae
h. Transitioning participants whose ADEs or SAEs have not resolved within 30 days of study completion or early termination to local clinical care
i. Promptly terminating study treatment in female participants who would become pregnant during the trial.

III.F.1.b **Definitions**

An Adverse Event (AE) is any untoward physical or psychological occurrence in a human subject participating in research. An AE can be any unfavorable and unintended event, including an abnormal laboratory finding, symptom, or disease associated with the research or the use of a medical investigational test article. An AE does not necessarily have to have a causal relationship with the research (Ref: VHA Handbook 1200.05, May 2, 1012).

In this trial, an Adverse Reaction (AR) is defined as an AE for which there is evidence the AE is clearly attributed to the Exogen device. A Suspected Adverse Reaction (SAR) is defined as an AE for which there is evidence to suggest the AE is possibly attributed to use of the Exogen device.

Treatment-emergent Adverse Events will be any event not present prior to the initiation of the study treatment, or any event already present that worsens in either intensity or frequency following exposure to the study treatment.

The severity of each Adverse Event will be graded according to the following scale:

- Mild (Grade 1): Asymptomatic or mild symptoms; clinical or diagnostic observations only; intervention not indicated.
- Moderate (Grade 2): Minimal, local, or noninvasive intervention (*e.g.*, packing, cautery) indicated; limiting age-appropriate instrumental activities of daily living (ADL).
- Severe (Grade 3): Medically significant but not immediately life-threatening; hospitalization or prolongation of hospitalization may be indicated; disabling; limiting self-care ADL.
- Life Threatening (Grade 4): In view of investigator or sponsor, participant is at an immediate risk of death; urgent intervention indicated.
- Death Related (Grade 5)

An Unanticipated Adverse Device Effect (UADE) means any serious adverse effect on health or safety or any life-threatening problem or death caused by, or associated with, a device, if that effect, problem or death was not previously identified in nature, severity, or degree of incidence in the investigational plan or application, or any other or any other unanticipated serious problem associated with a device that relates to the rights, safety, or welfare of subjects.

All AEs/UADEs with a reasonable causal relationship to the investigative test article should be considered at least "reasonably attributable." A definite relationship does not need to be established.

For the purpose of this study Serious Adverse Events (SAE) are a subset of AEs that:

a. Results in death;
b. Are life-threatening;
c. Requires inpatient hospitalization or prolongation of existing hospitalization;
d. Results in persistent or significant incapacity or substantial disruption of the ability to conduct normal functions;
e. Results in a congenital anomaly/birth defect.
f. Note: Important medical events that may not result in death, be life-threatening, or require hospitalization may be considered serious when, based upon appropriate medical judgment, they may jeopardize the patient or subject and may require medical or surgical intervention to prevent one of the outcomes listed in the SAE definition.

Events of Special Concerns include 1) suicide, 2) death due to an unusual circumstance, 3) death, great bodily harm, or significant property damage by a study participant, and 4) medical errors. The following table summarizes reporting requirements for site investigators.

III.F.2 AE, SAE, ADE, and UADE Monitoring and Reporting Requirements

Event Type	Every 30-day Follow-up	Expedited Reporting to Sponsor	Review of Study CRF	Local IRB Reporting
Adverse Reactions (ARs)	Yes	No	Yes	No
Suspected Adverse Reactions (SAR)	Yes	No	Yes	No
Unanticipated Adverse Device Effects (UADEs)†	Yes	Yes, 72 hours	Yes	Yes
Non-Device-Related Adverse Events	No	No	Yes	No
Events of Special Concerns	Sponsor may direct follow up frequency when applicable	Yes, immediately	Yes	Yes
Serious Adverse Events (SAEs)†	Yes	Yes, 72 hours	Yes	Yes, if unanticipated

†Note: UADEs and SAEs will be followed up to 30 days past study completion or early termination

UADEs and SAEs require expedited reporting by site investigators. Expedited reporting is defined as the completion and submission of the UADE or SAE form to the study's SharePoint website within 72 clock-hours of the site investigator or site coordinator becoming aware of either an SAE or UADE.

The site investigator is responsible for grading AEs/SAEs/ADEs/UADEs' severity, determining attributability to the study intervention, and determining whether any event is anticipated. The CSPCRPCC is responsible for evaluating all UADE and SAE reports in a timely manner. In addition, a Clinical Events Committee (CEC) will be formed to adjudicate UADEs and SAEs that occur in the study. The committee involves the study Chair, a physician from the CSPCC, a GCP and safety monitor from the CSPCC, and the AE Specialist from the CSPCRPCC. This committee is independent from the site investigators.

UADEs and SAEs that are related to the investigative treatment and determined to be unexpected will be reported to the Food and Drug Administration (FDA), the CSPCC Director, and the site investigators after review by the Study Chair, the CSPCC Director, and the AE Specialist. Site investigators should follow local regulations to inform their IRB of Record. All participants presenting to the VA medical center will be provided treatment for all AEs/SAEs/ADEs that occur during the study period.

III.F.3 Reporting of Events of Special Concerns

Site investigators will generate a written report to describe Events of Special Concerns immediately upon becoming aware of such events. The report will be promptly forwarded to the CEC for review and notification of CSP Central Office. Events of Special Concerns are defined in section III.F.1.b.

III.G Data Monitoring Committee

This study will assemble a Data Monitoring Committee (DMC) who will review the progress of the study, including patient recruitment, participant compliance,completeness of follow-up, data quality, protocol deviations, and safety. The DMC may also choose to implement a formal interim monitoring rule to monitor efficacy. The DMC will review any protocol modifications recommended by the Executive Committee. The DMC will establish criteria for study termination and make recommendations to the Director, Clinical Sciences Research and Development (CSR&D), through the Director, Hines VA CSPCC, as to whether the study should continue or be terminated. Should the DMC recommend study termination, the CSPCRPCC will promptly notify the FDA. Interim unblinded progress reports will be provided to the DMC by the study biostatistician. The DMC shall be composed of four experts in the study's subject matter fields, specifically, rheumatic diseases, clinical trials, biostatistics, and ethics.

III.G.1 Reporting AEs, UADEs and SAEs to the Data Monitoring Committee (DMC)

The Hines CSPCC will generate tabulations of all AEs, UADEs and SAEs for the DMC on an annual basis or on a more frequent schedule if requested by the DMC.

III.H Females and Pregnancy Testing

Females of childbearing potential who are breastfeeding, pregnant or plan to become pregnant during the study or within 3 months following the last study treatment will be excluded from participating in the study for safety reasons. Women who are surgically sterile or have been post-menopausal for at least 2 years are not considered to be of childbearing potential. Females of childbearing potential will have a urine pregnancy test performed at the Screening

visit 1.0 and the Day 0, Beginning of Period 3 part of visit 5.0, and must be willing to use a reliable form of medically acceptable contraception throughout their participation in the study.

III.I Women, Minorities and Children

Male and female individuals of all races will be recruited and no minorities will be excluded. To maximize the homogeneity of the participant group in terms of speech/language characteristics, participants must have spoken English as their primary language. Children will be excluded from participating.

IV. REFERENCES

1. Kothari, M., et al., Fixed-flexion radiography of the knee provides reproducible joint space width measurements in osteoarthritis. Eur Radiol, 2004. **14**(9): p. 1568-73.
2. Iranpour-Boroujeni T, L.J., Lynch J, Nevitt M, Duryea J, A new method to measure anatomic knee alignment: A tool for large studies of OA? , in Osteoarthritis Cartilage2012. p. S20-21.
3. Hawker, G.A., et al., Measures of adult pain: Visual Analog Scale for Pain (VAS Pain), Numeric Rating Scale for Pain (NRS Pain), McGill Pain Questionnaire (MPQ), Short-Form McGill Pain Questionnaire (SF-MPQ), Chronic Pain Grade Scale (CPGS), Short Form-36 Bodily Pain Scale (SF-36 BPS), and Measure of Intermittent and Constant Osteoarthritis Pain (ICOAP). Arthritis Care Res (Hoboken), 2011. **63 Suppl 11**: p. S240-52.
4. Bond, M., et al., Responsiveness of the OARSI-OMERACT osteoarthritis pain and function measures. Osteoarthritis Cartilage, 2012. **20**(6): p. 541-7.
5. Gurkan, I., et al., Modification of osteoarthritis in the guinea pig with pulsed low-intensity ultrasound treatment. Osteoarthritis Cartilage, 2010. **18**(5): p. 724-33.
6. Cook, S.D., et al., Improved cartilage repair after treatment with low-intensity pulsed ultrasound. Clin Orthop Relat Res, 2001(391 Suppl): p. S231-43.
7. Loyola-Sanchez, A., J. Richardson, and N.J. MacIntyre, Efficacy of ultrasound therapy for the management of knee osteoarthritis: a systematic review with meta-analysis. Osteoarthritis Cartilage, 2010. **18**(9): p. 1117-26.
8. Peterfy, C., et al., Comparison of fixed-flexion positioning with fluoroscopic semi-flexed positioning for quantifying radiographic joint-space width in the knee: test-retest reproducibility. Skeletal Radiol, 2003. **32**(3): p. 128-32.
9. Eckstein, F., et al., Quantitative MRI measures of cartilage predict knee replacement: a case-control study from the Osteoarthritis Initiative. Ann Rheum Dis, 2012.
10. Brandt, K.D., et al., Effects of doxycycline on progression of osteoarthritis: results of a randomized, placebo-controlled, double-blind trial. Arthritis Rheum, 2005. **52**(7): p. 2015-25.
11. Pham, T., et al., OMERACT-OARSI initiative: Osteoarthritis Research Society International set of responder criteria for osteoarthritis clinical trials revisited. Osteoarthritis Cartilage, 2004. **12**(5): p. 389-99.
12. Cameron, K.L., et al., Incidence of physician-diagnosed osteoarthritis among active duty United States military service members. Arthritis Rheum, 2011. **63**(10): p. 2974-82.
13. Hochberg, M.C., et al., American College of Rheumatology 2012 recommendations for the use of nonpharmacologic and pharmacologic therapies in osteoarthritis of the hand, hip, and knee. Arthritis Care Res (Hoboken), 2012. **64**(4): p. 455-74.

14. Grodzinsky, A.J., et al., Cartilage tissue remodeling in response to mechanical forces. Annu Rev Biomed Eng, 2000. **2**: p. 691-713.
15. Heath, C.A. and S.R. Magari, Mini-review: Mechanical factors affecting cartilage regeneration in vitro. Biotechnol Bioeng, 1996. **50**(4): p. 430-7.
16. Nolte, P.A., et al., Low-intensity pulsed ultrasound in the treatment of nonunions. J Trauma, 2001. **51**(4): p. 693-702; discussion 702-3.
17. Leung, K.S., et al., Low intensity pulsed ultrasound stimulates osteogenic activity of human periosteal cells. Clin Orthop Relat Res, 2004(418): p. 253-9.
18. Zhang, Z.J., et al., The effects of pulsed low-intensity ultrasound on chondrocyte viability, proliferation, gene expression and matrix production. Ultrasound Med Biol, 2003. **29**(11): p. 1645-51.
19. Nishikori, T., et al., Effects of low-intensity pulsed ultrasound on proliferation and chondroitin sulfate synthesis of cultured chondrocytes embedded in Atelocollagen gel. J Biomed Mater Res, 2002. **59**(2): p. 201-6.
20. Parvizi, J., et al., Low-intensity ultrasound stimulates proteoglycan synthesis in rat chondrocytes by increasing aggrecan gene expression. J Orthop Res, 1999. **17**(4): p. 488-94.
21. Yang, K.H., et al., Exposure to low-intensity ultrasound increases aggrecan gene expression in a rat femur fracture model. J Orthop Res, 1996. **14**(5): p. 802-9.
22. Miyamoto, K., et al., Exposure to pulsed low intensity ultrasound stimulates extracellular matrix metabolism of bovine intervertebral disc cells cultured in alginate beads. Spine (Phila Pa 1976), 2005. **30**(21): p. 2398-405.
23. Iwashina, T., et al., Low-intensity pulsed ultrasound stimulates cell proliferation and proteoglycan production in rabbit intervertebral disc cells cultured in alginate. Biomaterials, 2006. **27**(3): p. 354-61.
24. Choi, B.H., et al., Low-intensity ultrasound stimulates the viability and matrix gene expression of human articular chondrocytes in alginate bead culture. J Biomed Mater Res A, 2006. **79**(4): p. 858-64.
25. Min, B.H., et al., Effects of low-intensity ultrasound (LIUS) stimulation on human cartilage explants. Scand J Rheumatol, 2006. **35**(4): p. 305-11.
26. Zhang, Z.J., et al., The influence of pulsed low-intensity ultrasound on matrix production of chondrocytes at different stages of differentiation: an explant study. Ultrasound Med Biol, 2002. **28**(11-12): p. 1547-53.
27. Gooch, K.J., et al., IGF-I and mechanical environment interact to modulate engineered cartilage development. Biochem Biophys Res Commun, 2001. **286**(5): p. 909-15.
28. Huang, M.H., et al., Ultrasound effect on level of stress proteins and arthritic histology in experimental arthritis. Arch Phys Med Rehabil, 1999. **80**(5): p. 551-6.
29. Bhatia, R., V.K. Sobti, and K.S. Roy, Gross and histopathological observations on the effects of therapeutic ultrasound in experimental acute chemical arthritis in calves. Zentralbl Veterinarmed A, 1992. **39**(3): p. 168-73.

30. Singh KI, S.V., Roy KS, Gross and histomorphological effects of therapeutic ultrasound (1 Watt/Cm^2) in exprerimental acute traumatic arthritis in donkeys. Journal of Equine Veterinary Science, 1997. **17**(3): p. 150-155.
31. Malizos, K.N., et al., Low-intensity pulsed ultrasound for bone healing: an overview. Injury, 2006. **37 Suppl 1**: p. S56-62.
32. Eckstein, F. and W. Wirth, Quantitative cartilage imaging in knee osteoarthritis. Arthritis, 2011. **2011**: p. 475684.
33. Bellamy, N., et al., Validation study of WOMAC: a health status instrument for measuring clinically important patient relevant outcomes to antirheumatic drug therapy in patients with osteoarthritis of the hip or knee. J Rheumatol, 1988. **15**(12): p. 1833-40.
34. Loyola-Sanchez, A., et al., Effect of low-intensity pulsed ultrasound on the cartilage repair in people with mild to moderate knee osteoarthritis: a double-blinded, randomized, placebo-controlled pilot study. Arch Phys Med Rehabil, 2012. **93**(1): p. 35-42.
35. Lawrence, R.C., et al., Estimates of the prevalence of arthritis and other rheumatic conditions in the United States. Part II. Arthritis Rheum, 2008. **58**(1): p. 26-35.
36. Kotlarz, H., et al., Insurer and out-of-pocket costs of osteoarthritis in the US: evidence from national survey data. Arthritis Rheum, 2009. **60**(12): p. 3546-53.
37. Hootman, J.M. and C.G. Helmick, Projections of US prevalence of arthritis and associated activity limitations. Arthritis Rheum, 2006. **54**(1): p. 226-9.
38. Kuo, A.C., et al., Microfracture and bone morphogenetic protein 7 (BMP-7) synergistically stimulate articular cartilage repair. Osteoarthritis Cartilage, 2006. **14**(11): p. 1126-35.
39. Malinin, T., H.T. Temple, and B.E. Buck, Transplantation of osteochondral allografts after cold storage. J Bone Joint Surg Am, 2006. **88**(4): p. 762-70.
40. Wasiak, J., C. Clar, and E. Villanueva, Autologous cartilage implantation for full thickness articular cartilage defects of the knee. Cochrane Database Syst Rev, 2006. **3**: p. CD003323.
41. Ochi, M., et al., Articular cartilage repair using tissue engineering technique--novel approach with minimally invasive procedure. Artif Organs, 2004. **28**(1): p. 28-32.
42. Rubin, C., et al., The use of low-intensity ultrasound to accelerate the healing of fractures. J Bone Joint Surg Am, 2001. **83-A**(2): p. 259-70.
43. Tang, C.H., et al., Ultrasound stimulates cyclooxygenase-2 expression and increases bone formation through integrin, focal adhesion kinase, phosphatidylinositol 3-kinase, and Akt pathway in osteoblasts. Mol Pharmacol, 2006. **69**(6): p. 2047-57.
44. Irrechukwu, O.N., et al., Magnetic resonance studies of macromolecular content in engineered cartilage treated with pulsed low-intensity ultrasound. Tissue Eng Part A, 2011. **17**(3-4): p. 407-15.

45. Yoon, D.M. and J.P. Fisher, Chondrocyte signaling and artificial matrices for articular cartilage engineering. Adv Exp Med Biol, 2006. **585**: p. 67-86.

46. Salter, D.M., et al., Differential responses of chondrocytes from normal and osteoarthritic human articular cartilage to mechanical stimulation. Biorheology, 2002. **39**(1-2): p. 97-108.

47. Huang, M.H., et al., Effects of sonication on articular cartilage in experimental osteoarthritis. J Rheumatol, 1997. **24**(10): p. 1978-84.

48. Singh, K.I., V.K. Sobti, and K.S. Roy, Gross and histomorphological effects of therapeutic ultrasound (1 Watt/cm2) in experimental acute traumatic arthritis in donkeys. Journal of Equine Veterinary Science, 1997. **17**(3): p. 150-155.

49. Park, S.R., et al., The effect of sonication on simulated osteoarthritis. Part II: alleviation of osteoarthritis pathogenesis by 1 MHz ultrasound with simultaneous hyaluronate injection. Ultrasound Med Biol, 2005. **31**(11): p. 1559-66.

50. Bendele, A.M. and J.F. Hulman, Spontaneous cartilage degeneration in guinea pigs. Arthritis Rheum, 1988. **31**(4): p. 561-5.

51. Hunter, D.J. and F. Eckstein, From joint anatomy to clinical outcomes in osteoarthritis and cartilage repair: summary of the fifth annual osteoarthritis imaging workshop. Osteoarthritis Cartilage, 2011. **19**(11): p. 1263-9.

52. Zhang, Y., et al., Fluctuation of knee pain and changes in bone marrow lesions, effusions, and synovitis on magnetic resonance imaging. Arthritis Rheum, 2011. **63**(3): p. 691-9.

53. Yusuf, E., et al., Do knee abnormalities visualised on MRI explain knee pain in knee osteoarthritis? A systematic review. Ann Rheum Dis, 2011. **70**(1): p. 60-7.

54. Eckstein, F., D. Burstein, and T.M. Link, Quantitative MRI of cartilage and bone: degenerative changes in osteoarthritis. NMR Biomed, 2006. **19**(7): p. 822-54.

55. Eckstein, F., A. Guermazi, and F.W. Roemer, Quantitative MR imaging of cartilage and trabecular bone in osteoarthritis. Radiol Clin North Am, 2009. **47**(4): p. 655-73.

56. Wirth, W., et al., Comparison of 1-year vs 2-year change in regional cartilage thickness in osteoarthritis results from 346 participants from the Osteoarthritis Initiative. Osteoarthritis Cartilage, 2011. **19**(1): p. 74-83.

57. Le Graverand, M.P., et al., Change in regional cartilage morphology and joint space width in osteoarthritis participants versus healthy controls: a multicentre study using 3.0 Tesla MRI and Lyon-Schuss radiography. Ann Rheum Dis, 2010. **69**(1): p. 155-62.

58. Eckstein, F., et al., Double echo steady state magnetic resonance imaging of knee articular cartilage at 3 Tesla: a pilot study for the Osteoarthritis Initiative. Ann Rheum Dis, 2006. **65**(4): p. 433-41.

59. Eckstein, F., et al., Quantitative MRI measures of cartilage predict knee replacement: a case-control study from the Osteoarthritis Initiative. Ann Rheum Dis, 2012.

60. Wirth, W. and F. Eckstein, A technique for regional analysis of femorotibial cartilage thickness based on quantitative magnetic resonance imaging. IEEE Trans Med Imaging, 2008. **27**(6): p. 737-44.

61. Wirth, W., et al., Regional analysis of femorotibial cartilage loss in a subsample from the Osteoarthritis Initiative progression subcohort. Osteoarthritis Cartilage, 2009. **17**(3): p. 291-7.

62. Hunter, D.J., et al., Evolution of semi-quantitative whole joint assessment of knee OA: MOAKS (MRI Osteoarthritis Knee Score). Osteoarthritis Cartilage, 2011. **19**(8): p. 990-1002.

63. Peterfy, C.G., et al., Whole-Organ Magnetic Resonance Imaging Score (WORMS) of the knee in osteoarthritis. Osteoarthritis Cartilage, 2004. **12**(3): p. 177-90.

64. Hunter, D.J., et al., The reliability of a new scoring system for knee osteoarthritis MRI and the validity of bone marrow lesion assessment: BLOKS (Boston Leeds Osteoarthritis Knee Score). Ann Rheum Dis, 2008. **67**(2): p. 206-11.

65. Kurtz, S., et al., Projections of primary and revision hip and knee arthroplasty in the United States from 2005 to 2030. J Bone Joint Surg Am, 2007. **89**(4): p. 780-5.

66. Mankin, H.J., A.Z. Thrasher, and D. Hall, Biochemical and metabolic characteristics of articular cartilage from osteonecrotic human femoral heads. J Bone Joint Surg Am, 1977. **59**(6): p. 724-8.

67. Brandt, K.D., et al., Which is the best radiographic protocol for a clinical trial of a structure modifying drug in patients with knee osteoarthritis? J Rheumatol, 2002. **29**(6): p. 1308-20.

68. Mazzuca, S.A., et al., Knee pain reduces joint space width in conventional standing anteroposterior radiographs of osteoarthritic knees. Arthritis Rheum, 2002. **46**(5): p. 1223-7.

69. Eckstein, F., et al., Precision of 3.0 Tesla quantitative magnetic resonance imaging of cartilage morphology in a multicentre clinical trial. Ann Rheum Dis, 2008. **67**(12): p. 1683-8.

70. Eckstein, F., W. Wirth, and M.C. Nevitt, Recent advances in osteoarthritis imaging-the Osteoarthritis Initiative. Nat Rev Rheumatol, 2012.

71. Clegg, D.O., D.J. Reda, and M. Abdellatif, Comparison of sulfasalazine and placebo for the treatment of axial and peripheral articular manifestations of the seronegative spondylarthropathies: a Department of Veterans Affairs cooperative study. Arthritis Rheum, 1999. **42**(11): p. 2325-9.

72. Clegg, D.O., et al., Comparison of sulfasalazine and placebo in the treatment of psoriatic arthritis. A Department of Veterans Affairs Cooperative Study. Arthritis Rheum, 1996. **39**(12): p. 2013-20.

73. Clegg, D.O., et al., Comparison of sulfasalazine and placebo in the treatment of ankylosing spondylitis. A Department of Veterans Affairs Cooperative Study. Arthritis Rheum, 1996. **39**(12): p. 2004-12.

74. Clegg, D.O., et al., Comparison of sulfasalazine and placebo in the treatment of reactive arthritis (Reiter's syndrome). A Department of Veterans Affairs Cooperative Study. Arthritis Rheum, 1996. **39**(12): p. 2021-7.

75. Clegg, D.O., et al., Glucosamine, chondroitin sulfate, and the two in combination for painful knee osteoarthritis. N Engl J Med, 2006. **354**(8): p. 795-808.
76. Sawitzke, A.D., et al., The effect of glucosamine and/or chondroitin sulfate on the progression of knee osteoarthritis: a report from the glucosamine/chondroitin arthritis intervention trial. Arthritis Rheum, 2008. **58**(10): p. 3183-91.
77. Sawitzke, A.D., et al., Clinical efficacy and safety of glucosamine, chondroitin sulphate, their combination, celecoxib or placebo taken to treat osteoarthritis of the knee: 2-year results from GAIT. Ann Rheum Dis, 2010. **69**(8): p. 1459-64.
78. Javaid, M., et al., Individual MRI and radiographic features of knee OA in subjects with unilateral knee pain: Health ABC study. Arthritis Rheum, 2012.
79. Eckstein, F., et al., How do short-term rates of femorotibial cartilage change compare to long-term changes? Four year follow-up data from the osteoarthritis initiative. Osteoarthritis Cartilage, 2012.
80. Kellgren, J.H. and J.S. Lawrence, Radiological assessment of osteo-arthrosis. Ann Rheum Dis, 1957. **16**(4): p. 494-502.
81. Hochberg, M.C., et al., The American College of Rheumatology 1991 revised criteria for the classification of global functional status in rheumatoid arthritis. Arthritis Rheum, 1992. **35**(5): p. 498-502.
82. Hochberg, M.C., et al., Design and conduct of clinical trials in osteoarthritis: preliminary recommendations from a task force of the Osteoarthritis Research Society. J Rheumatol, 1997. **24**(4): p. 792-4.
83. Cibere, J., et al., Reliability of the knee examination in osteoarthritis: effect of standardization. Arthritis Rheum, 2004. **50**(2): p. 458-68.
84. Laslett, L.L., et al., Zoledronic acid reduces knee pain and bone marrow lesions over 1 year: a randomised controlled trial. Ann Rheum Dis, 2012. **71**(8): p. 1322-8.
85. Kraus, V.B., et al., Application of biomarkers in the development of drugs intended for the treatment of osteoarthritis. Osteoarthritis Cartilage, 2011. **19**(5): p. 515-42.
86. Dam, E.B., et al., Identification of progressors in osteoarthritis by combining biochemical and MRI-based markers. Arthritis Res Ther, 2009. **11**(4): p. R115.
87. Deberg, M.A., et al., One-year increase of Coll 2-1, a new marker of type II collagen degradation, in urine is highly predictive of radiological OA progression. Osteoarthritis Cartilage, 2005. **13**(12): p. 1059-65.
88. Cibere, J., et al., Association of biomarkers with pre-radiographically defined and radiographically defined knee osteoarthritis in a population-based study. Arthritis Rheum, 2009. **60**(5): p. 1372-80.
89. Dworkin, R.H., et al., Outcome measures in placebo-controlled trials of osteoarthritis: responsiveness to treatment effects in the REPORT database. Osteoarthritis Cartilage, 2011. **19**(5): p. 483-92.

90. Simon, R., R.E. Wittes, and S.S. Ellenberg, Randomized phase II clinical trials. Cancer Treat Rep, 1985. **69**(12): p. 1375-81.
91. Gibbons JD, O.R., Sobel M, Selecting and Ordering Populations: A New Statistical Methodology. Philadelphia: Society for Industrial and Applied Mathematics. 1977, New York: John Wiley & Sons.
92. Gibbons JD, O.I., Sobel M, Introduction to Ranking and Selection. American Statistician, 1979. **33**(4): p. 185-195.
93. Reginster, J.Y., et al., Efficacy and safety of strontium ranelate in the treatment of knee osteoarthritis: results of a double-blind, randomised placebo-controlled trial. Ann Rheum Dis, 2013. **72**(2): p. 179-86.
94. Molenberghs, G.a.K., M G, Missing Data in Clinical Studies. 2007, Chichester, UK: John Wiley and Sons Ltd.
95. Hedeker, D., Gibbons RD, Longitudinal Data Analysis. 2006, Hoboken (NJ): John Wiley & Sons.
96. Chao, J., et al., Inflammatory characteristics on ultrasound predict poorer longterm response to intraarticular corticosteroid injections in knee osteoarthritis. J Rheumatol, 2010. **37**(3): p. 650-5.
97. Ibrahim SA, H.B., Hannon MJ, Kresevic D, Long J, Kwoh CK, Willingness and access to joint replacement among black patients with knee osteoarthritis: A randomized, controlled intervention. Arthritis Rheum, 2013. **Accepted for Publication**.
98. Wildi, L.M., et al., Chondroitin sulphate reduces both cartilage volume loss and bone marrow lesions in knee osteoarthritis patients starting as early as 6 months after initiation of therapy: a randomised, double-blind, placebo-controlled pilot study using MRI. Ann Rheum Dis, 2011. **70**(6): p. 982-9.
99. Spirito, A., et al., Sources of site differences in the efficacy of a multi-site clinical trial: the treatment of SSRI resistant depression in adolescents. J Consult Clin Psychol, 2009. **77**(3): p. 439-50.

V. APPENDICES (not embedded within protocol)

V.A **CCTA 0008 MR Imaging Protocol**

V.B **CCTA 0008 X-Ray Imaging Protocol**

V.C **Knee Examination Protocol**
