# Supplementary material for: Effect of Pulsed Low-Intensity Ultrasonography on Symptom Relief and Tibiofemoral Articular Cartilage Thickness Among Veterans Affairs Enrollees With Knee Osteoarthritis: A Randomized Clinical Trial
Source: JAMA Netw Open. 2022 Mar 8;5(3):e220632. doi: 10.1001/jamanetworkopen.2022.0632 (PMC8905392; doi:10.1001/jamanetworkopen.2022.0632)
Supplement: Supplement 2. — eTable 1. Average Analgesia by Group eTable 2. NSAID Use During the Trial eFigure. Mean WOMAC Pain Subscale Over Time by Treatment Group [file jamanetwopen-e220632-s002.pdf]

Supplementary Online Content

Sawitzke AD, Jackson CG, Carlson K, et al. Effect of pulsed low-intensity ultrasonography on symptom relief and tibiofemoral articular cartilage thickness among Veterans Affairs enrollees with knee osteoarthritis: a randomized clinical trial. *JAMA Netw Open*. 2022;5(3):e220632. doi:10.1001/jamanetworkopen.2022.0632

eTable 1. Average Analgesia by Group

eTable 2. NSAID Use During the Trial

eFigure. Mean WOMAC Pain Subscale Over Time by Treatment Group

This supplementary material has been provided by the authors to give readers additional information about their work.

© 2022 Sawitzke AD et al. *JAMA Network Open.*

eTable 1. Average Analgesia by Group

Visit	Acetaminophen			Tramadol		
	PLIUS*	SHAM*	P-value	PLIUS*	SHAM*	P-value
**7**	1114.9 (590.4)	904.7 (764.8)	0.58	77.2 (116.0)	89.4 (76.7)	0.87
**8**	840.1 (728.4)	890.2 (880.2)	0.86	81.3 (82.7)	90.5 (65.9)	0.83
**10**	893.6 (821.3)	736.1 (697.0)	0.67	82.7 (100.3)	90.5 (45.3)	0.88
**12**	798.3 (720.9)	817.7 (736.7)	0.94	67.0 (91.1)	84.3 (70.7)	0.71
**14**	826.7 (843.0)	731.7 (844.7)	0.75	64.6 (77.3)	73.3 (88.2)	0.83
**16**	1010.4 (697.9)	852.7 (985.4)	0.66	67.9 (72.3)	38.0 (39.1)	0.38
**18**	1075.5 (650.3)	736.9 (944.6)	0.29	67.9 (76.9)	80.2 (75.3)	0.79

*-Average daily dosage in mg (sd)

© 2022 Sawitzke AD et al. *JAMA Network Open.*

eTable 2. NSAID Use During the Trial

NSAID Used During the Study*	Treatment Group				Total		P-value
	PLIUS		SHAM				
	N	%	N	%	N	%	
**No**	37	55.2	39	60.0	76	57.6	0.60
**Yes**	30	44.8	26	40.0	56	42.4	

*-Has the participant ever taken a NSAID-Nonsteroidal Anti-Inflammatory Drug during the study.

© 2022 Sawitzke AD et al. *JAMA Network Open.*

eFigure. Mean WOMAC Pain Subscale Over Time by Treatment Group

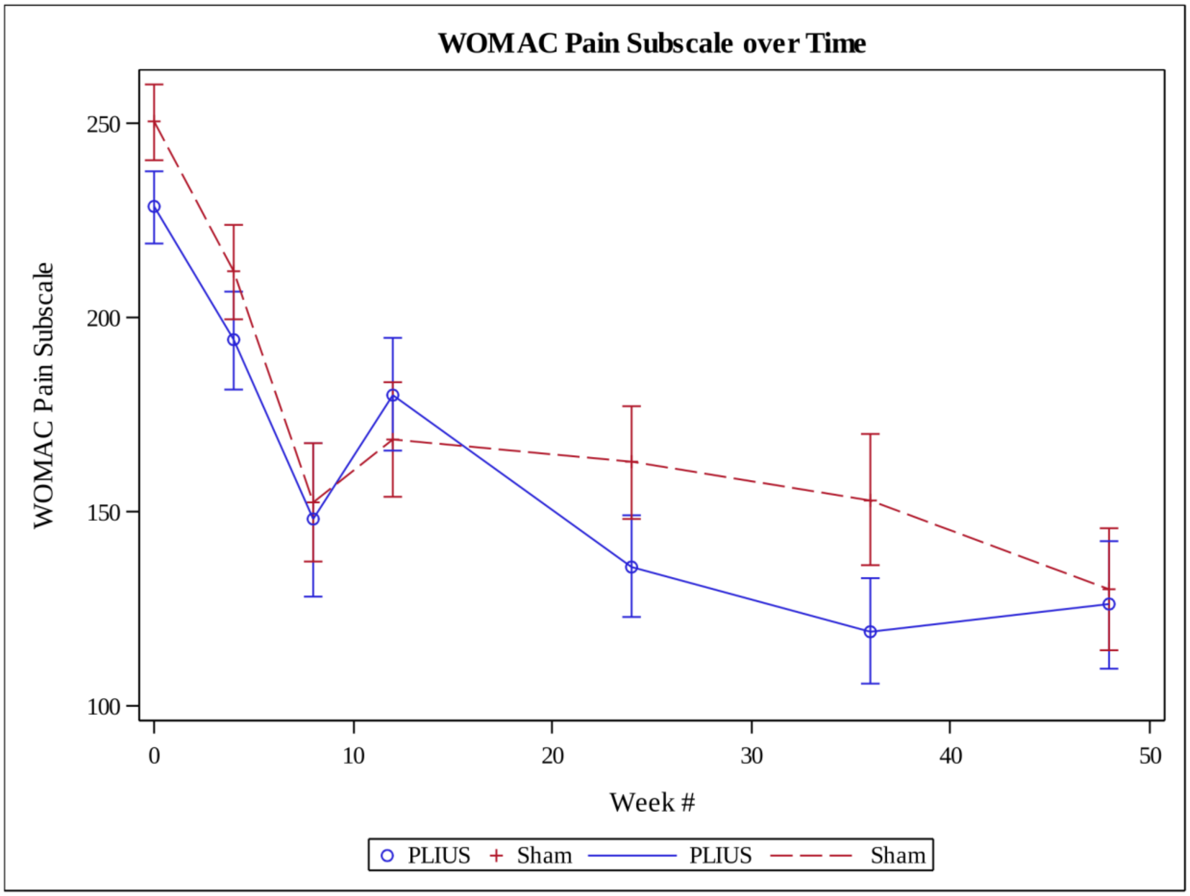

The mean WOMAC pain subscore +/- 2 SD is shown by week of the trial. Larger numbers represent more pain.

© 2022 Sawitzke AD et al. *JAMA Network Open.*
