# Supplementary material for: Effect of Pulsed Low-Intensity Ultrasonography on Symptom Relief and Tibiofemoral Articular Cartilage Thickness Among Veterans Affairs Enrollees With Knee Osteoarthritis: A Randomized Clinical Trial
Source: JAMA Netw Open. 2022 Mar 8;5(3):e220632. doi: 10.1001/jamanetworkopen.2022.0632 (PMC8905392; doi:10.1001/jamanetworkopen.2022.0632)
Supplement: Supplement 3. — Data Sharing Statement [file jamanetwopen-e220632-s003.pdf]

Data Sharing Statement

Sawitzke. Effect of Pulsed Low-Intensity Ultrasonography on Symptom Relief and Tibiofemoral Articular Cartilage Thickness Among Veterans Affairs Enrollees With Knee Osteoarthritis. *JAMA Netw Open*. Published March 08, 2022. doi:10.1001/jamanetworkopen.2022.0632

Data
Data available: Yes
Data types: Deidentified participant data
How to access data: Kimberly.Carlson@va.gov
When available: With publication

Supporting Documents
Document types: None

Additional Information
Who can access the data: Anyone approved by committee
Types of analyses: All approved projects
Mechanisms of data availability: Deidentified data-sets may be shared when the purpose of use has been approved by the appropriate VA oversight committee and an agreement is in place that defines the limits of this use.
